# Synthesis and Structure–Activity Relationship Studies of Derivatives of the Dual Aromatase–Sulfatase Inhibitor 4-{[(4-Cyanophenyl)(4*H*-1,2,4-triazol-4-yl)amino]methyl}phenyl sulfamate

**DOI:** 10.1002/cmdc.201300015

**Published:** 2013-03-11

**Authors:** L W Lawrence Woo, Paul M Wood, Christian Bubert, Mark P Thomas, Atul Purohit, Barry V L Potter

**Affiliations:** [a] Medicinal Chemistry, Department of Pharmacy and Pharmacology, University of BathClaverton Down, Bath BA2 7AY (UK); [b] Diabetes, Endocrinology & Metabolism, Imperial College London, Section of Investigative Medicine6th Floor, Commonwealth Building (6N2B), Hammersmith Hospital, Du Cane Road, London W12 0NN (UK)

**Keywords:** aromatase, breast cancer, dual inhibitors, endocrine therapy, steroid sulfatase

## Abstract

4-{[(4-Cyanophenyl)(4*H*-1,2,4-triazol-4-yl)amino]methyl}phenyl sulfamate and its *ortho*-halogenated (F, Cl, Br) derivatives are first-generation dual aromatase and sulfatase inhibitors (DASIs). Structure–activity relationship studies were performed on these compounds, and various modifications were made to their structures involving relocation of the halogen atom, introduction of more halogen atoms, replacement of the halogen with another group, replacement of the methylene linker with a difluoromethylene linker, replacement of the *para*-cyanophenyl ring with other ring structures, and replacement of the triazolyl group with an imidazolyl group. The most potent in vitro DASI discovered is an imidazole derivative with IC_50_ values against aromatase and steroid sulfatase in a JEG-3 cell preparation of 0.2 and 2.5 nm, respectively. The parent phenol of this compound inhibits aromatase with an IC_50_ value of 0.028 nm in the same assay.

## Introduction

In modern medicine, it is common practice to treat many diseases concurrently with several drugs that target multiple biological targets via different mechanisms of action in order to achieve the desirable therapeutic outcome concertedly. To this end, an increasing number of clinically established drugs are reformulated to give a fixed-dose dual-component medicine as an alternative to co-administering the agents as two individual drugs. While this approach is an obvious choice for attaining a combined therapy, an attractive and more elegant strategy is to design a single drug that inhibits more than one biological target. In fact, the design of single agents that act against multiple biological targets is of increasing interest and prominence. In recent years, an increasing volume of work has been published exemplifying the successful use of this strategy.^1^–^11^

**Figure 1:**
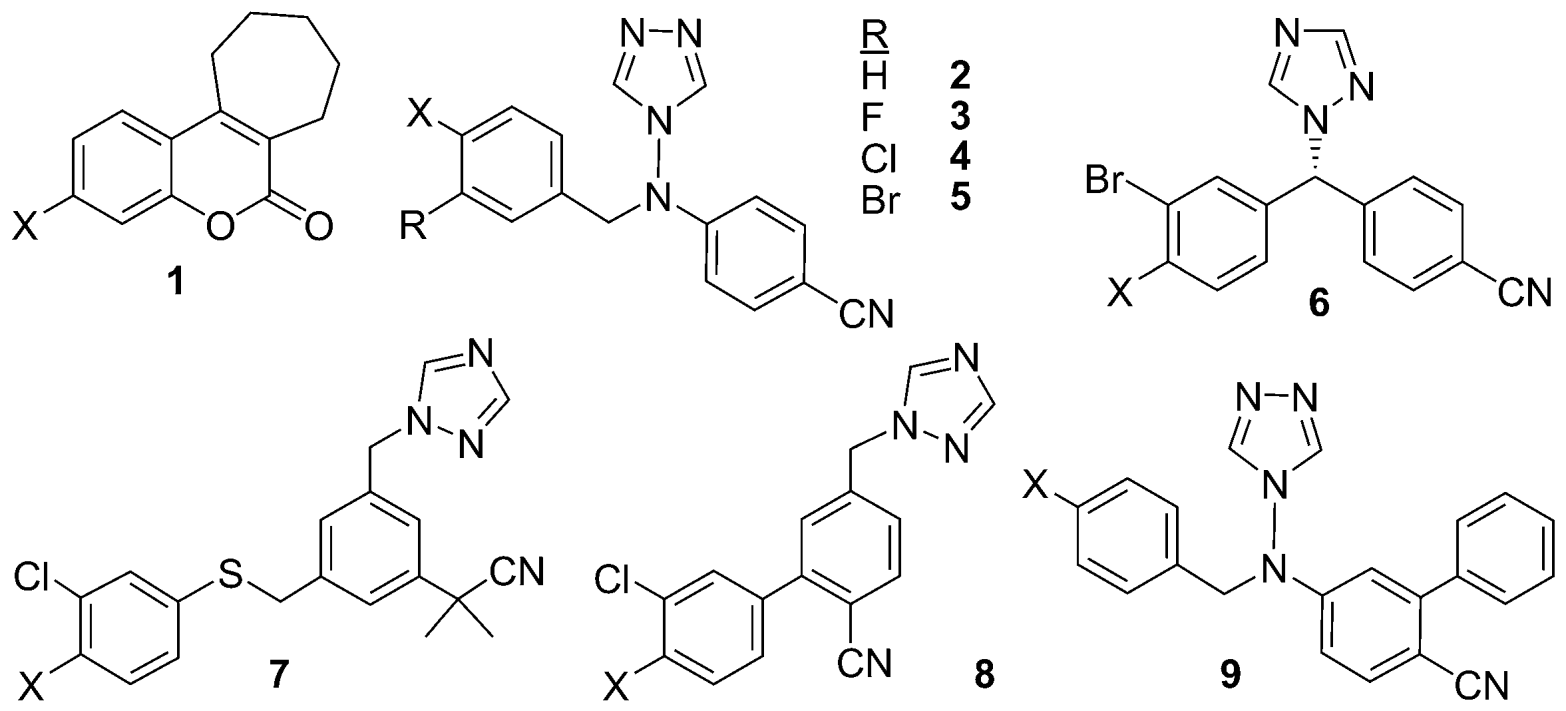
Structures of nonsteroidal steroid sulfatase inhibitor **1** (STX64, Irosustat) and various structural classes of dual aromatase–sulfatase inhibitor (DASI) **2**–**9**; X=OSO_2_NH_2_.

Breast cancer is a devastating disease that affects many women from different age groups. When the disease is first diagnosed, about two-thirds of cases are classified as hormone-dependent, in which the growth and development of tumours are fuelled by oestrogens. Endocrine therapy is an effective form of treatment for breast cancer of this type. The current gold standard for treating patients with hormone-dependent breast cancer is a selective oestrogen receptor modulator (SERM) such as tamoxifen, which blocks the action of oestrogens on the oestrogen receptor. In addition, the inhibition of aromatase, which leads to a decrease in the biosynthesis of oestrogens, is well established as an effective form of endocrine treatment for breast cancer. The three aromatase inhibitors (AIs) currently in clinical use for treating patients with advanced hormone-dependent breast cancer (HDBC) are letrozole, anastrozole, and exemestane. Various clinical trials in the last decade have even advocated the use of these AIs in an earlier clinical setting for treating primary breast cancer.^12^–^18^ However, inhibition of aromatase is not the only strategy available for decreasing the production of oestrogens. There is evidence that inhibition of steroid sulfatase (STS), the enzyme that converts the biologically inactive oestrone sulfate to oestrone,^19^ may render significant oestrogen deprivation in patients treated with an STS inhibitor. The nonsteroidal compound **1** (STX64, Irosustat, Figure 1) is the first STS inhibitor entered into clinical trials.^20^, ^21^

Because both aromatase and STS are involved in the synthesis of biologically active oestrogens, it has been reasoned that concurrent inhibition of these two enzymes in patients with HDBC may lead to a more comprehensive oestrogen deprivation and hence a better response to endocrine therapy. Our research group has pioneered the approach of designing dual aromatase and sulfatase inhibitors (DASIs) as a single agent for inhibiting aromatase and STS concurrently. To date, we have developed five structural classes of nonsteroidal DASIs. Briefly: 1) derivatives of the nonsteroidal AI 4-{(4-bromobenzyl)-[1,2,4]triazol-4-ylamino}benzonitrile (e.g., **2**–**5**, Figure 1);^22^–^25^ 2) derivatives of letrozole (e.g., **6**, Figure 1);^26^–^28^ 3) derivatives of anastrozole (e.g., **7**, Figure 1);^29^ 4) derivatives based on a biphenyl template (e.g., **8**, Figure 1);^30^ and 5) a series of compounds with a hybrid structure of experimental DASIs (e.g., **9**, Figure 1).^31^ Herein we report the further expansion of the series of DASIs **2**–**5**. Inhibitory activities of new candidates against aromatase and STS were evaluated in JEG-3 cells, and structure–activity relationships (SAR) are discussed.

## Results and Discussion

### Chemistry

**Scheme 1:**
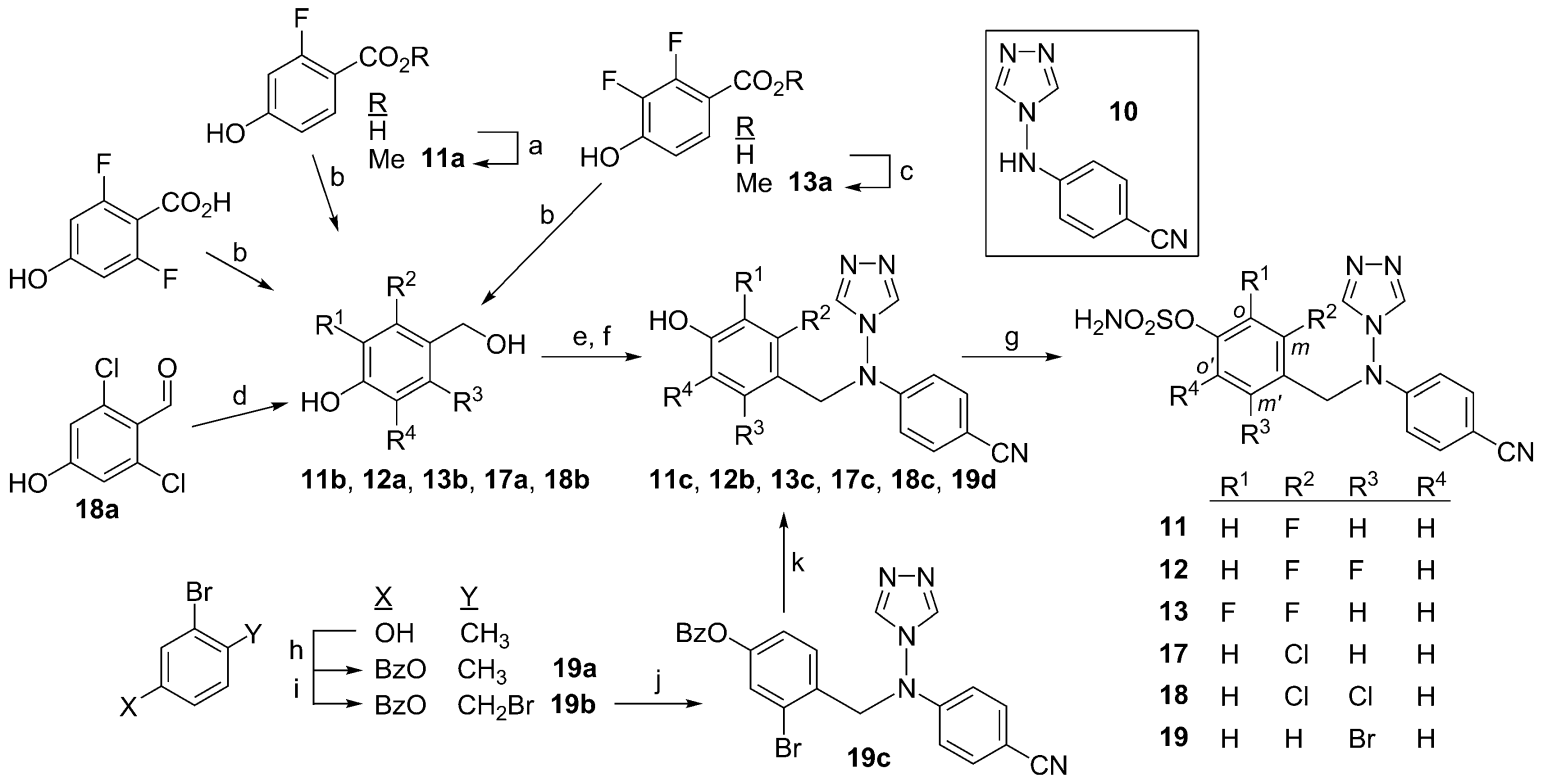
Synthesis of compounds **11**–**13** and **17**–**19**. *Reagents and conditions*: a) MeOH, HCl (cat.), reflux, 78 %; b) LiAlH_4_, THF, RT, 76–94 %; c) SOCl_2_, MeOH, reflux, 100 %; d) NaBH_4_, EtOH/H_2_O, RT, 93 %; e) SOCl_2_, RT; f) 10, K_2_CO_3_, DMF, RT, 15–100 %; g) H_2_NSO_2_Cl, DMA, 0 °C→RT, 55–95 %; h) BzCl, py, RT, 75 %; i) NBS, (BzO)_2_, CCl_4_, reflux, 51 %; j) **10**, NaH, DMF, RT, 85 %; k) KOH, MeOH, 52 %.

The preparation of 4-[(4-cyanophenyl)amino]-4*H*-1,2,4-triazole (**10**, Scheme 1) was carried out according to Okada et al.^32^ All of the novel sulfamates and phenols described herein were prepared according to the schemes described below. The final compounds and intermediates were characterised by standard analytical methods, and the purity of the compounds tested in vitro was evaluated by HPLC.

The synthesis of sulfamates **11**–**13**, **17**–**19** is outlined in Scheme 1. Following formation of the requisite benzyl alcohols **11 b**, **12 a**, **13 b**, **17 a**, **18 b**, the final three steps of the synthesis were identical for each of the sulfamates. The appropriate benzyl alcohol was converted into the corresponding benzyl chloride with thionyl chloride, and this was allowed to react with **10** in *N*,*N*-dimethylformamide (DMF) in the presence of potassium carbonate to give phenols **11 c**, **12 b**, **13 c**, **17 c,** and **18 d**, respectively. The phenols were converted into their corresponding sulfamates with excess sulfamoyl chloride according to the conditions described by Okada et al.^33^ Benzyl alcohols **11 b** and **13 b** were obtained from 2-fluoro- and 2,3-difluoro-4-hydroxybenzoic acids, respectively, following conversion into their methyl esters (with methanol/cat. HCl for **11 b** and methanol/SOCl_2_ for **13 b**) and reduction of these with lithium aluminium hydride in tetrahydrofuran. From 2,6-difluoro-4-hydroxybenzoic acid, benzyl alcohol **12 a** was obtained directly following reduction with LiAlH_4_ in THF.

For the synthesis of compound **19**, 3-bromo-4-methylphenol was protected as the benzoate **19 a** and subsequently brominated with *N*-bromosuccinimide (NBS) under radical conditions ((BzO)_2_, CCl_4_) to afford **19 b**. Benzyl bromide **19 b** was used to alkylate the deprotonated form of **10** to give the benzoate **19 c**, which was hydrolysed with potassium hydroxide in methanol to the phenol **19 d**. Phenol **19 d** was treated with excess sulfamoyl chloride in *N*,*N*-dimethylacetamide (DMA) to give **19**.

**Scheme 2:**
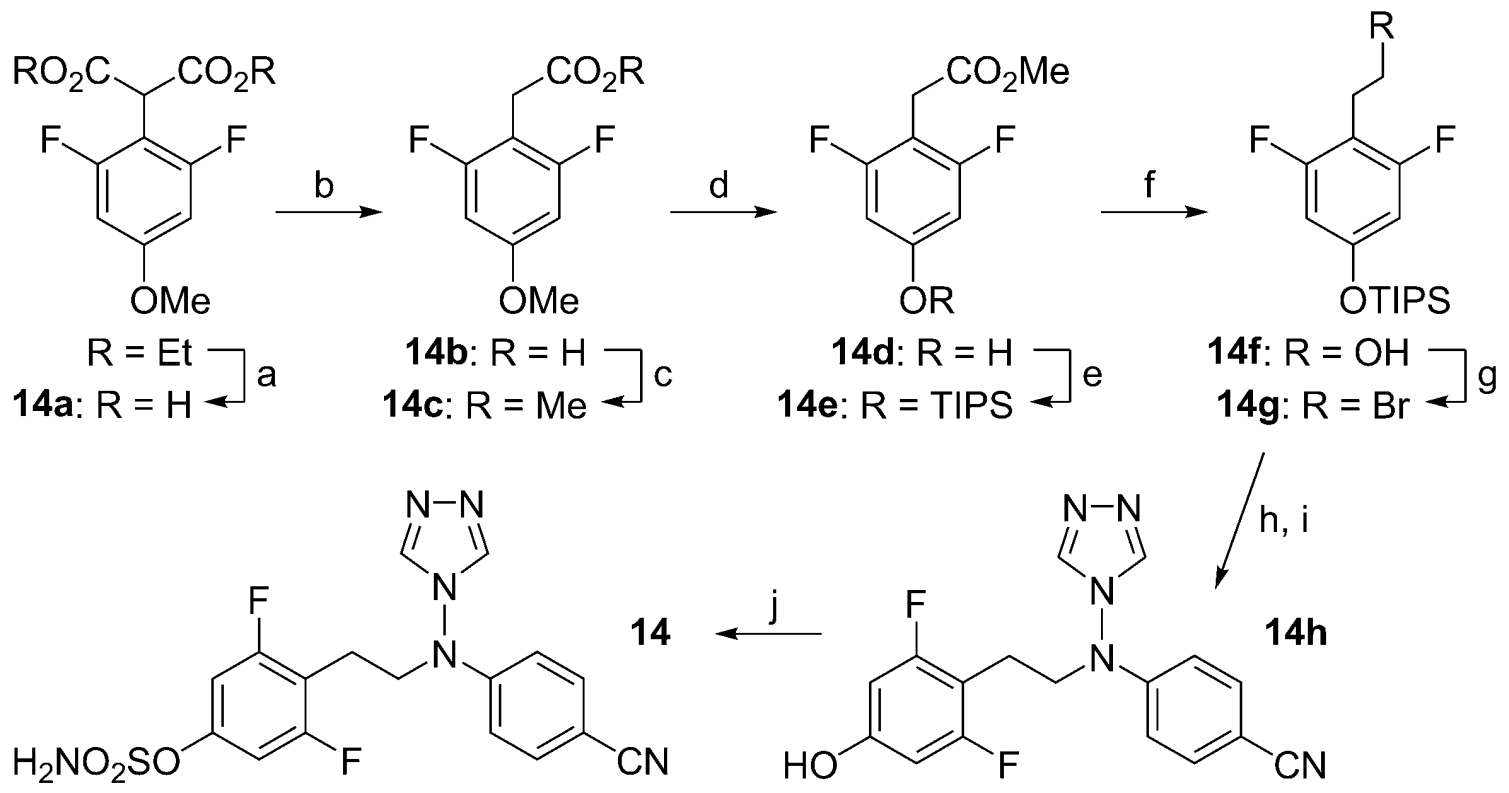
Synthesis of compound **14**. *Reagents and conditions*: a) 3 m NaOH, reflux, 88 %; b) 160 °C, 93 %; c) MeOH, HCl (conc.), reflux, 92 %; d) BBr_3_, CH_2_Cl_2_, −78 °C→RT, 77 %; e) TIPSCl, imidazole, DMF, RT, 91 %; f) LiAlH_4_, THF, 0 °C, 62 %; g) PPh_3_, CBr_4_, CH_2_Cl_2_, 0 °C→RT, 67 %; h) **10**, NaH, DMF, RT; i) TBAF, THF, RT, 41 %; j) H_2_NSO_2_Cl, DMA, 0 °C→RT, 53 %.

The route to sulfamate **14** starting from diethyl-2-(2,6-difluoro-4-methoxyphenyl)malonic acid,^34^ which was itself synthesised by the reaction of diethyl malonate with 4-bromo-3,5-difluoroanisole, is shown in Scheme 2. The diester was hydrolysed to diacid **14 a** with 3 m sodium hydroxide, and this decarboxylated following heating at 160 °C to give **14 b**. The acid was converted into methyl ester **14 c**, and the phenol was demethylated following treatment with boron tribromide in dichloromethane and then re-protected with triisopropylsilyl chloride (TIPSCl) to furnish **14 e**. The methyl ester was reduced with LiAlH_4_ to give alcohol **14 f**, which was converted into the corresponding bromide following treatment with PPh_3_/CBr_4_ in dichloromethane. This was subsequently used to alkylate the sodium hydride generated anion of **10**. Removal of the TIPS protecting group was achieved with tetra-*n*-butylammonium fluoride (TBAF) in THF, and the resulting phenol **14 h** was converted into sulfamate **14** under the conditions previously described.

**Scheme 3:**
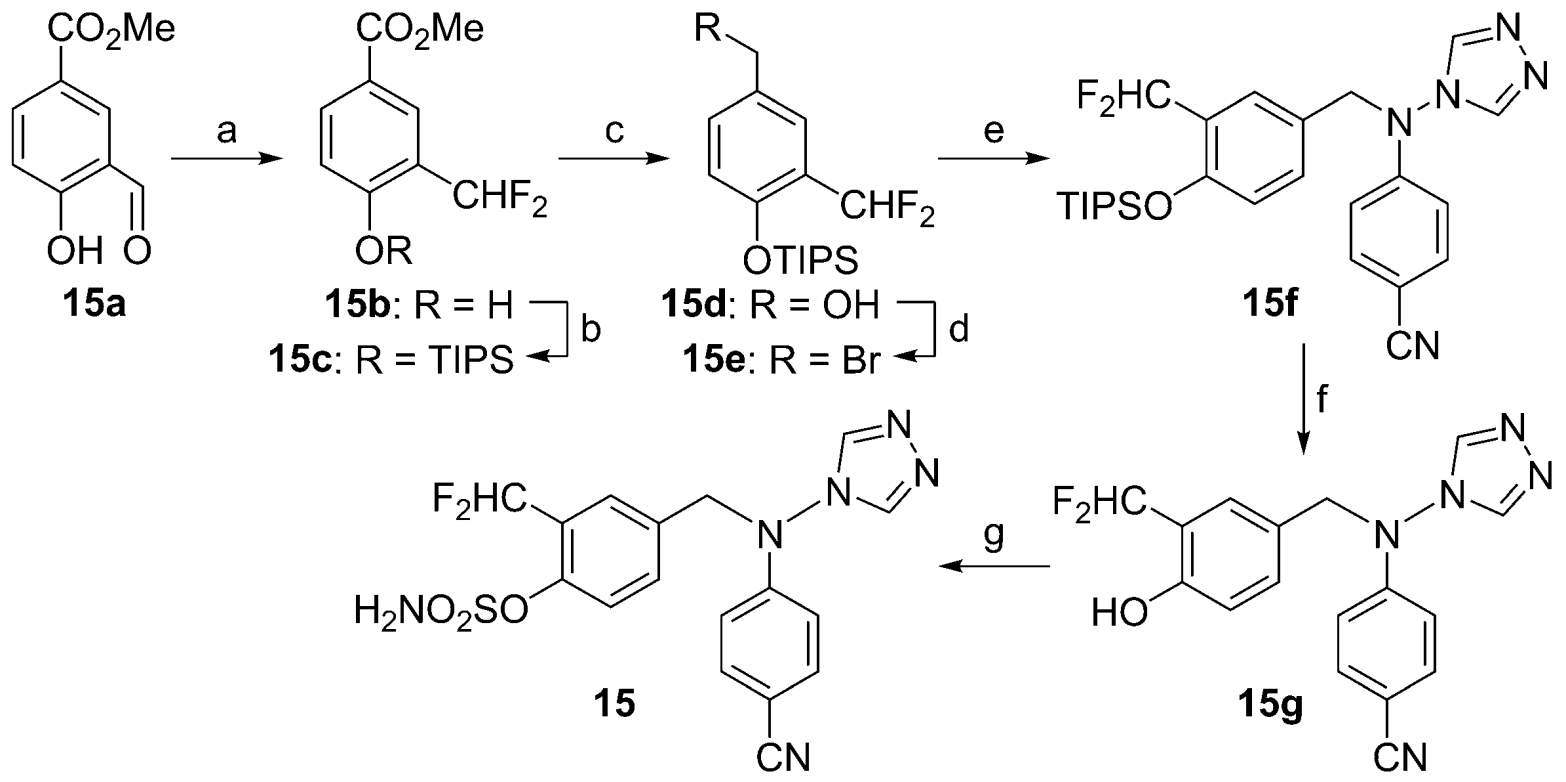
Synthesis of compound **15**. *Reagents and conditions*: a) Deoxo-Fluor®, EtOH, CH_2_Cl_2_, RT, 75 %; b) TIPSCl, imidazole, DMF, RT, 99 %; c) LiBH_4_, B(OMe)_3_, Et_2_O, RT, 67 %; d) PBr_3_, CH_2_Cl_2_, 0 °C→RT, 76 %; e) **10**, NaH, DMF, 0 °C→RT, 74 %; f) TBAF, THF, RT, 95 %; g) H_2_NSO_2_Cl, DMA, 0 °C→RT, 90 %.

Sulfamate **15** was prepared in seven steps (Scheme 3) from 3-formyl-4-hydroxybenzoic acid methyl ester **15 a**, which was prepared from methyl 4-hydroxybenzoate as described by Hofsløkken and Skattebol.^35^ Starting material **15 a** was fluorinated with Deoxo-Fluor® to give phenol **15 b**, which was then TIPS-protected to furnish **15 c**. Reduction of the ester was successfully achieved with lithium borohydride, and the resulting benzyl alcohol was converted into the corresponding benzyl bromide with phosphorus tribromide in dichloromethane. This was subsequently used to alkylate the sodium hydride generated anion of **10** to furnish **15 f**. Removal of the TIPS protecting group was carried out with TBAF in THF, and the resulting phenol **15 g** was converted into sulfamate **15** under the conditions previously described.

**Scheme 4:**
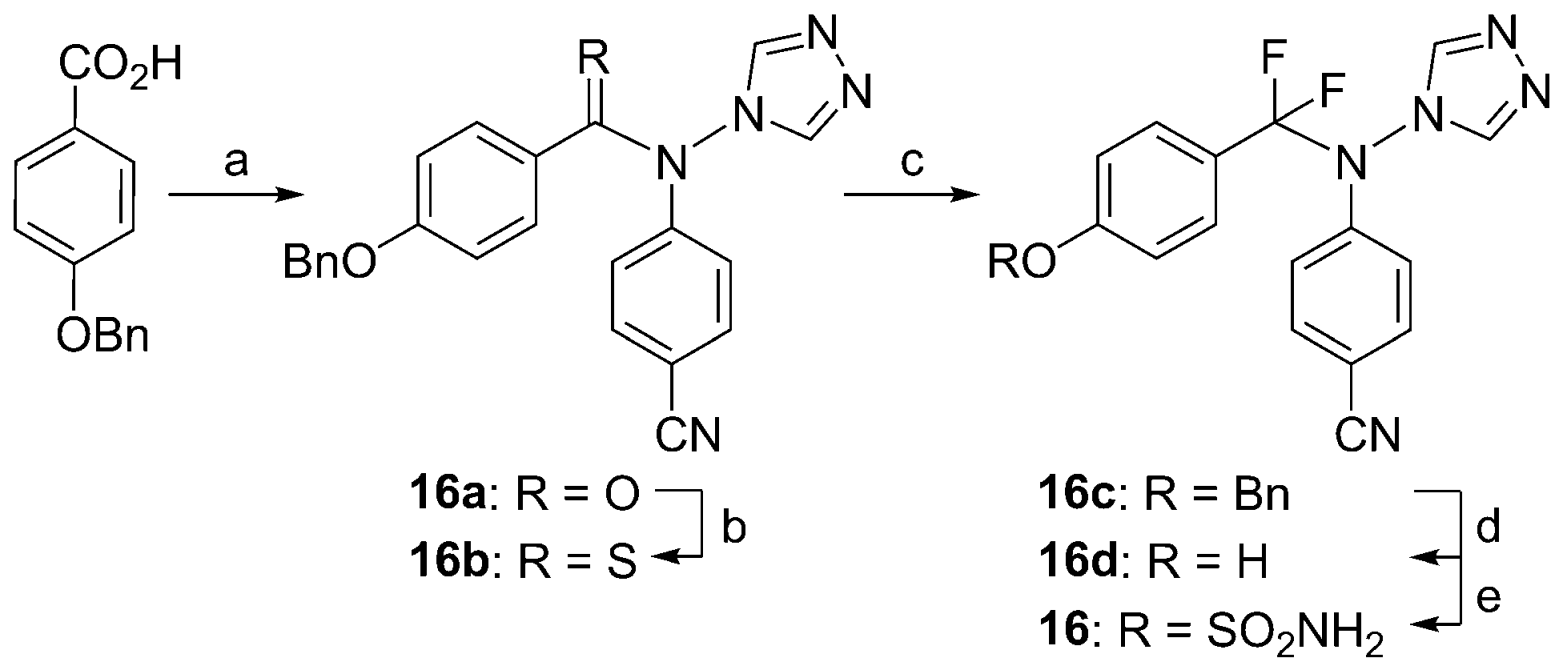
Synthesis of compound **16**. *Reagents and conditions*: a) SOCl_2_, reflux, then **10**, Et_3_N, CH_2_Cl_2_, RT, 67 %; b) Lawesson’s reagent, xylenes, reflux, 56 %; c) Deoxo-Fluor®, SbCl_3_, CH_2_Cl_2_, RT, 71 %; d) H_2_, Pd/C (10 %), THF/MeOH, RT, 61 %; e) H_2_NSO_2_Cl, DMA, 0 °C→RT, 76 %.

Sulfamate **16** was prepared from 4-benzyloxybenzoic acid according to the route illustrated in Scheme 4. Following preparation of the acid chloride with thionyl chloride, reaction with **10** gave amide **16 a**. The amide was converted into the corresponding thioamide with Lawesson’s reagent in xylenes at reflux, and this was fluorinated with Deoxo-Fluor® in the presence of catalytic antimony trichloride to give **16 c**.^36^ Deprotection of the phenol was achieved by catalytic hydrogenation with palladium on charcoal to give **16 d**, and the formation of the corresponding sulfamate was achieved using the conditions previously described to give **16**.

**Scheme 5:**
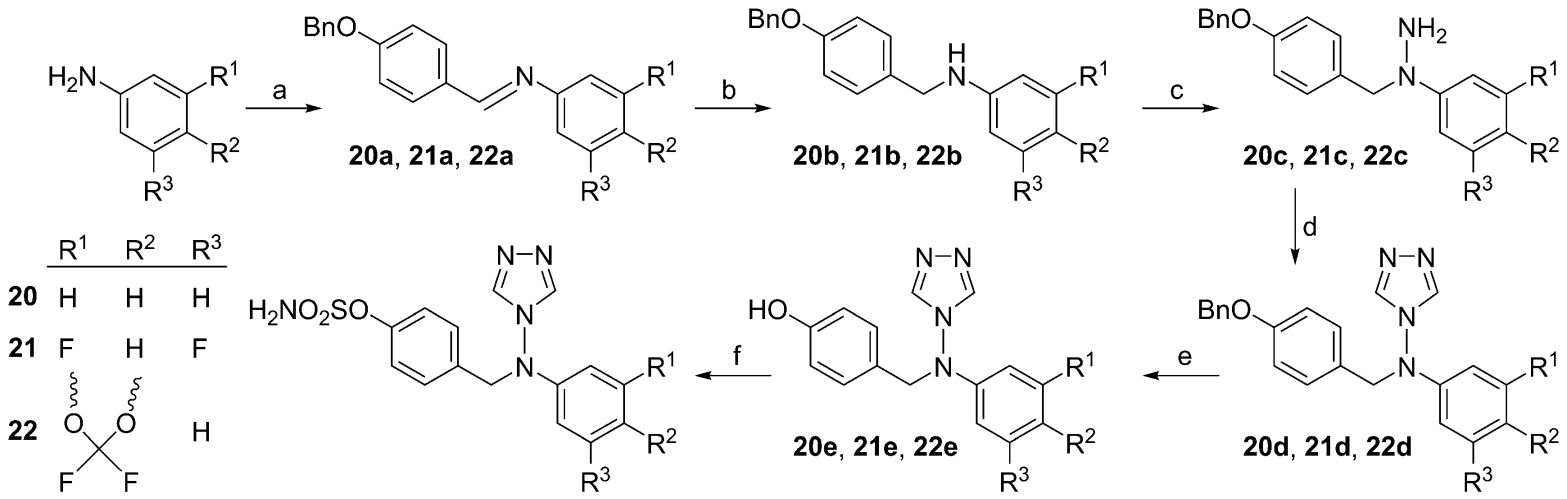
Synthesis of compound **20**, **21** and **22**. *Reagents and conditions*: a) 4-benzyloxybenzaldehyde, *p*-TsOH, EtOH, reflux, 78–85 %; b) NaBH_4_, EtOH/H_2_O, 0 °C→RT, 94–96 %; c) NaNO_2_, H_2_O, CHCl_3_, 2 m H_2_SO_4_, 0 °C, then LiAlH_4_, THF, RT, 72–86 %; d) dimethylformamide diazine dihydrochloride, py, reflux, 53–70 %; e) H_2_, Pd/C (5 %), THF/MeOH, RT, 74–92 %; f) H_2_NSO_2_Cl, DMA, 0 °C, 27–75 %.

The trio of sulfamates **20**, **21**, and **22** were prepared according to the route described in Scheme 5 by starting from commercially available aniline, 5-amino-2,2-difluoro-1,3-benzodioxole, and 3,5-difluoroaniline, respectively. Each amine was condensed with 4-benzyoxybenzaldehyde to give imines **20 a**, **21 a**, and **22 a**, which were subsequently reduced with sodium borohydride to give amines **20 b**, **21 b**, and **22 b**. The triazole ring was constructed stepwise, firstly by treating the requisite amines with sodium nitrite and then with LiAlH_4_ to give hydrazines **20 c**, **21 c**, and **22 c**. This was followed by treatment of the hydrazines with dimethylformamide diazine dihydrochloride in pyridine at reflux^37^ to furnish **20 d**, **21 d**, and **22 d**. The benzyl group was removed by catalytic hydrogenation with palladium on charcoal, and finally the phenol was sulfamoylated under the conditions described above to give compounds **20**, **21**, and **22**.

**Scheme 6:**
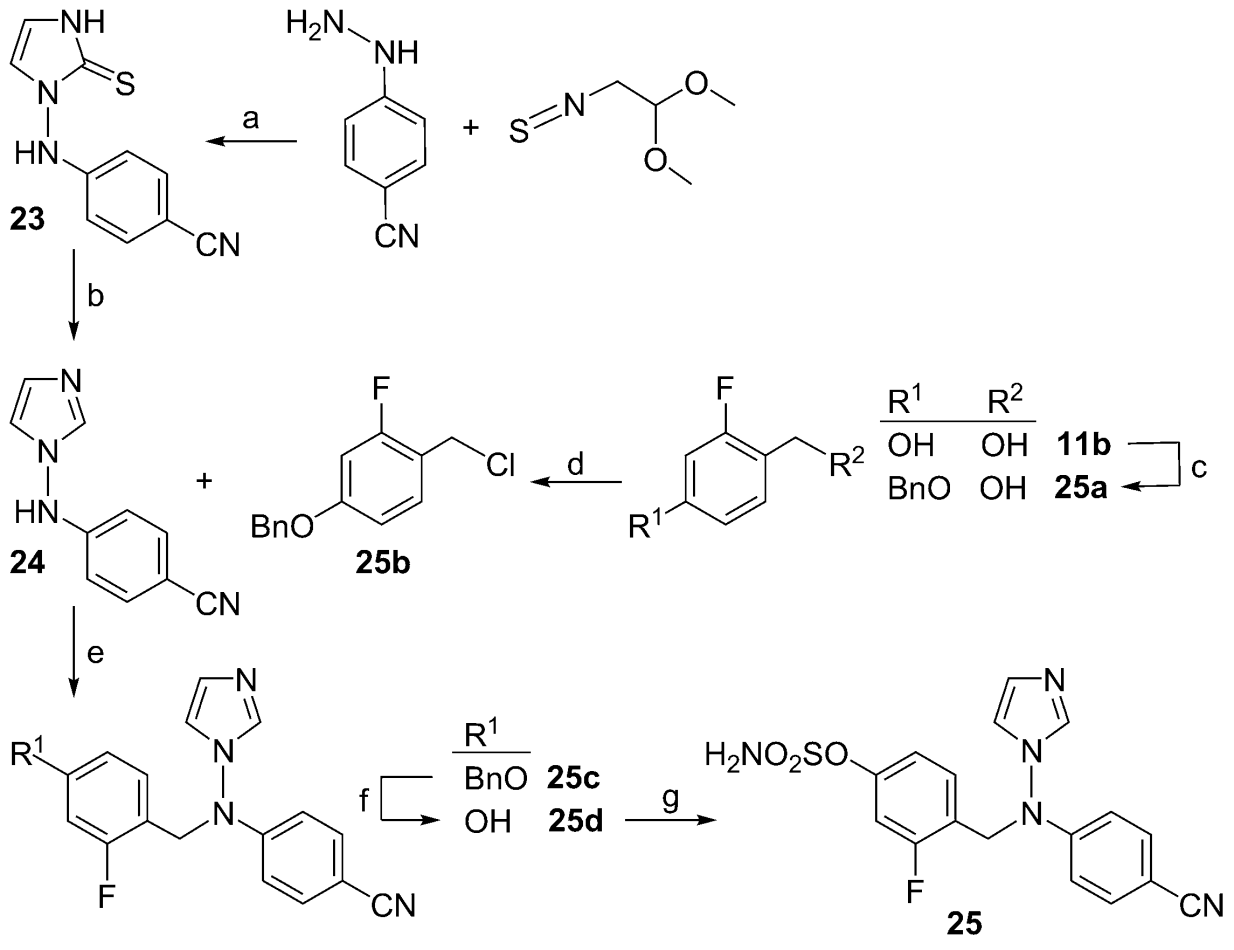
Synthesis of **25**. *Reagents and conditions*: a) AcOH/H_2_O (10:1), 100 °C, 1 h, 80 %; b) AcOH, 50 % H_2_O_2_, 0 °C, 59 %; c) BnBr, K_2_CO_3_, DMF, RT, 84 %; d) SOCl_2_, CH_2_Cl_2_, RT, 85 %; e) NaH, DMF, RT, 85 %; f) H_2_, Pd/C (5 %), THF/MeOH (1:1), 81 %; g) H_2_NSO_2_Cl, DMA, 0 °C→RT, 88 %.

The synthesis of compound **25** is shown in Scheme 6. 4-(1*H*-Imidazol-1-ylamino)benzonitrile (**24**) was prepared in two steps following a patent procedure.^38^ Commercially available 4-cyanophenylhydrazine hydrochloride was allowed to react with 2-isothiocyanato-1,1-dimethoxyethane in acetic acid and water at 100 °C to give **23**, which was then treated with hydrogen peroxide in acetic acid to afford **24**. The benzyl chloride **25 b** was obtained by converting **11 b** into the benzyl ether **25 a** and subsequent treatment of **25 a** with thionyl chloride in dichloromethane. Benzyl chloride **25 b** was used to alkylate the sodium hydride generated anion of **24** to afford **25 c**. The benzyl group of **25 c** was removed by catalytic hydrogenation with palladium on charcoal, and finally the phenol **25 d** was converted into sulfamate **25** by using the conditions described above.

### Biological assays

The in vitro inhibition of aromatase and STS activity by each sulfamate was measured in a preparation of an intact monolayer of JEG-3 cells. The results are reported as IC_50_ values and are compared with those of published data for DASIs **2**–**5** (Table 1). We also tested the in vitro inhibition of aromatase by parent phenols in the same manner. The results are listed in Table 2 and are compared with published data for compounds **2 a**–**5 a**.

**Table 1:** In vitro inhibition of the aromatase and STS activity in a JEG-3 cell preparation by compounds 11–22 and 25; compounds 2–5 are included as reference.^23^

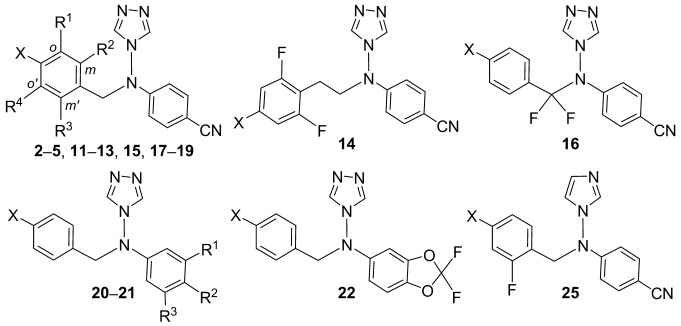						
Compd^[a]^	R^1^	R^2^	R^3^	R^4^	Aromatase IC_50_ [nm]	STS IC_50_ [nm]
**2**	H	H	H	H	100	227
**3**	F	H	H	H	12	40
**4**	Cl	H	H	H	2.3	20
**5**	Br	H	H	H	0.82	39
**11**	H	F	H	H	39±2.8	21±3.5
**12**	H	F	F	H	1.3±0.3	39±1
**13**	F	F	H	H	7.1±1.7	48±2
**14**	–	–	–	–	4.0±0.72	485±50.7
**15**	F_2_HC	H	H	H	4.4±0.1	60±2.4
**16**	–	–	–	–	1013±13	190±20
**17**	H	Cl	H	H	18±2	40±5
**18**	H	Cl	Cl	H	0.6±0.1	44±6
**19**	H	Br	H	H	2.6±0.3	29±2
**20**	H	H	H	–	2483±189	1557±116
**21**	F	H	F	–	760±110	45±5
**22**	–	–	–	–	4060±260	52±6.6
**25**	–	–	–	–	0.2±0.01	2.5±0.4

[a] X=OSO_2_NH_2_.

#### 1) Sulfamates

Although their potency against an individual enzyme is not as high as that observed for the single agent AI letrozole (IC_50_=0.89 nm, JEG-3),^25^ and STS inhibitor **1** (IC_50_=1.5 nm, JEG-3),^23^ reference compounds **2**–**5** are first-generation DASIs that show significant dual inhibition against aromatase and STS. A preliminary SAR study for these compounds established, among other things, that a halogen atom substituted at the *ortho* position to the sulfamate group contributes significantly to the biological activities observed for these compounds and that the sulfamate group positioned *para* to the methylene linker between the arylsulfamate motif and the 4-(4*H*-1,2,4-triazol-4-ylamino)benzonitrile moiety is crucial for potent STS inhibition.^23^ The SAR for this class of DASIs is further expanded in this work.

##### a) *m*-Halogenated derivatives 11, 17, and 19

The first modification made to DASIs **3**, **4**, and **5** was the relocation of their halogen atom from the *ortho* to the *meta* position to the sulfamate group to give derivatives **11** (*m*-fluoro), **17** (*m*-chloro), and **19** (*m*-bromo), respectively. As shown in Table 1, this modification decreases the ability of the derivatives to inhibit aromatase (fluorinated derivatives: **3**, IC_50_=12 nm vs. **11**, IC_50_=39 nm; chlorinated derivatives: **4**, IC_50_=2.3 nm vs. **17**, IC_50_=18 nm; brominated derivatives: **5**, IC_50_=0.82 nm vs. **19**, IC_50_=2.6 nm). These results suggest that substitution of a single halogen atom on the sulfamate-bearing phenyl ring in this class of DASIs is more effective for inhibiting aromatase if it is made at the *ortho* position to the sulfamate group. Like the *ortho*-halogenated derivatives **3**, **4**, and **5**, the fluoro derivative **11** is less potent than the chloro derivative **17**, which in turn is a weaker AI than the bromo derivative **19**. This ascending order of potency against aromatase observed might be related to the increase in lipophilicity of the compounds as the size of the halogen increases from fluorine to bromine. The relocation of a halogen from the *ortho* to the *meta* position to the sulfamate group does not affect the STS inhibitory activity significantly, as *meta*-halogenated compounds **11**, **17**, and **19** inhibit STS to a similar extent as their *ortho*-halogenated counterparts **3**, **4**, and **5** (fluorinated derivatives: **3**, IC_50_=40 nm vs. **11**, IC_50_=21 nm; chlorinated derivatives: **4**, IC_50_=20 nm vs. **17**, IC_50_=40 nm; brominated derivatives: **5**, IC_50_=39 nm vs. **19**, IC_50_=29 nm).

##### b) *m*,*m*′-Dihalogenated derivatives 12 and 18

Introduction of the same halogen atom at the remaining *meta* position to the sulfamate group of **11** (*m*-F) and **17** (*m*-Cl) to give the respective dihalogenated derivatives **12** (*m*-F, *m*′-F) and **18** (*m*-Cl, *m*′-Cl) significantly improves the inhibitory activity against aromatase (fluorinated derivatives: **11**, IC_50_=39 nm vs. **12**, IC_50_=1.3 nm; chlorinated derivatives: **17**, IC_50_=18 nm vs. **18**, IC_50_=0.6 nm). In addition, **12** and **18** are apparently even more potent AIs than the *ortho*-halogenated compounds **3** (*o*-F, IC_50_=12 nm) and **4** (*o*-Cl, IC_50_=2.3 nm). The higher lipophilicity anticipated for **12** and **18** might contribute to their greater potency observed for aromatase inhibition.

Unexpectedly, **12** and **18** show a similar potency to **3**, **4**, **11**, and **17** in their activities against STS. They all inhibit STS with IC_50_ values ranging between 20 and 44 nm. We have demonstrated repeatedly in previous SAR studies that the STS inhibitory activity of an aryl sulfamate can generally be increased by lowering the p*K*_a_ value, and hence improving the leaving group ability, of its parent phenol.^22^–^25^, ^27^–^31^, ^39^ Because of the electron-withdrawing effect on the sulfamate group exerted by the halogens at the *m*,*m*′-position, the p*K*_a_ values of parent phenols **12 b** and **18 c** are predicted to be between 0.4 and 1.0 log units lower than those of phenols **3 a**, **4 a**, **11 c**, and **17 c** (as calculated by ACD/Labs v11.02 and based on comparison between respective mono- and dihalogenated *para*-cresols). The fact that **12** and **18** are not more effective STS inhibitors than **3**, **4**, **11**, and **17**, as predicted, suggests their STS inhibitory activities observed might be influenced by other factors.

##### c) *o*,*m*-Difluorinated derivative 13

When the two fluoro atoms are substituted on the same side of the phenyl ring at the *ortho* and *meta* positions to the sulfamate group, the resulting derivative **13** (*o*-F, *m*-F, IC_50_=7 nm) inhibits aromatase about fivefold less effectively than **12** (*m*-F, *m*′-F, IC_50_=1.3 nm), although their STS inhibitory activities are similar (**12**, IC_50_=39 nm and **13**, IC_50_=48 nm). The dual inhibitory activity observed for **13** is similar to that of **3** (*o*-F, IC_50 arom._=12 nm and IC_50 STS_=40 nm). These results suggest that disubstitution of fluorine atoms is more effective for aromatase inhibition, but not for STS inhibition, if they are substituted at the *m*,*m*′-position rather than at the *o*,*m*-position on the sulfamate-bearing phenyl ring.

##### d) *o*-Difluoromethyl derivative 15

A previous SAR study had shown that 2-difluoromethyloestrone 3-*O*-sulfamate is a potent steroidal STS inhibitor with an IC_50_ value of 100 pm against STS in a placental microsome preparation.^40^ It was reasoned that the electron-withdrawing effect of the 2-fluoromethyl group, as well as the potential of the fluorine atoms to form hydrogen bonds with residues lining the catalytic active site of STS, are contributive factors to the high potency observed. To explore the effect of the difluoromethyl group on the STS and aromatase inhibitory activity of this class of DASIs, the *ortho*-fluoro atom of **3** was replaced with a difluoromethyl group. As shown in Table 1, the difluoromethyl group improves aromatase inhibition, as **15** (IC_50 arom._=4.4 nm) is threefold more potent than **3** as an AI. However, contrary to expectation, there is no apparent improvement on STS inhibition observed with **15**, showing an IC_50_ value of 60 nm against STS, compared with 40 nm for **3**. It is possible that the more flexible structure of this class of DASIs, in contrast to the more rigid backbone of a steroidal STS inhibitor, does not align the 2-difluoromethyl group effectively for favourable interactions within the STS active site.

##### e) Derivatives with an ethylene linker (14) and a difluoromethylene linker (16)

When the methylene linker between the arylsulfamate motif and the 4-(4*H*-1,2,4-triazol-4-ylamino)benzonitrile moiety of **12** was replaced by an ethylene group, such an extension decreases both the aromatase and STS inhibitory activities of the resulting derivative **14**, with the potency against STS more significantly impaired (**12**, IC_50 arom._=1.3 nm, IC_50 STS_=39 nm vs. **14**, IC_50 arom._=4 nm, IC_50 STS_=485 nm). A similar adverse effect on STS inhibition is observed when the methylene linker of **2**–**5** is replaced by either an ethylene or a propylene group; in contrast, however, a much improved or at least similar inhibition of aromatase was observed for the ethylene derivatives of **2**–**5**.^24^

In addition to elongating the methylene linker of **12**, the methylene linker of **2** was replaced with difluoromethylene to give **16**. We reasoned that this electron-withdrawing motif would, on the one hand, lower the p*K*_a_ of the parent phenol **16 d** and hence improve the ability of sulfamate **16** to inhibit STS; on the other hand, the two fluoro atoms should increase the lipophilicity of the derivative and hence make a relative improvement of the aromatase inhibition by **16** (clog *P* 0.875, cf. 0.179 for **12**, as calculated by ChemBioDraw Ultra v12.0.2). However, against some expectations, this modification significantly decreases the inhibitory activity of **16** (IC_50 arom._=1013 nm, IC_50 STS_=190 nm) against aromatase, but acts to maintain a level of STS inhibition similar to that observed for **2** (IC_50 arom._=100 nm, IC_50 STS_=227 nm). These results suggest that the difluoromethylene motif is tolerated by STS, but not by aromatase, when it replaces the methylene group as the linker between the aryl sulfamate motif and the 4-(4*H*-1,2,4-triazol-4-ylamino)benzonitrile moiety.

##### f) Replacement of the *para*-cyanophenyl ring of 2 with other entities (20, 21, and 22)

After varying the substitution pattern of halogens on the sulfamate-bearing aryl ring and modifying the alkylene linker in this class of compounds, the *para*-cyanophenyl ring of **2** was modified first by replacing its *para*-cyano group with a hydrogen atom to give **20**, and then by replacing the ring altogether with a 3,5-difluorophenyl ring or a 2,2-difluorobenzo[*d*][1,3]dioxol-5-yl moiety to give **21** and **22**, respectively.

As shown in Table 1, replacement of the cyano group of **2** with a hydrogen atom is clearly detrimental to dual inhibitory activity. The IC_50_ values of **20** for aromatase (2483 nm) and STS (1557 nm) inhibition are an order of magnitude higher than those observed for **2** (IC_50 arom._=100 nm, IC_50 STS_=227 nm). A previous SAR study on nonsteroidal AIs that are based on a biphenyl template (e.g., **8**, Figure 1) demonstrated that a cyano group substituted at the position on the phenyl ring *para* to a haem-ligating moiety, such as the triazolylmethyl group, is important for potent aromatase inhibition.^41^ Either the removal of the cyano group or the replacement of it with a fluorine or a chlorine atom leads to derivatives that are significantly weaker AIs.^41^ Docking studies on this class of biphenyl-based AIs into a homology model of human aromatase (PDB code: 1TQA) revealed that the cyano group might interact favourably with Ser478 of the active site through hydrogen bond interactions.^41^

In addition to its positive effect on aromatase inhibition, the *para*-cyano group is apparently also important for STS inhibition, as **20** is some sevenfold weaker as an inhibitor than **2**. It is possible that the *para*-cyano group of **2** functions as a hydrogen bond acceptor and interacts favourably with amino acid residue(s) lining the enzyme active site of STS, allowing a better binding affinity of **2** to the enzyme active site than **20**.

Replacement of the *para*-cyanophenyl group of **2** with a 3,5-difluorophenyl ring or a 2,2-difluorobenzo[*d*][1,3]dioxol-5-yl moiety produces much weaker AIs, although the ability of **21** (IC_50_=760 nm) to inhibit aromatase is better than that of **22** (IC_50_=4060 nm), suggesting that the 3,5-difluorophenyl ring interacts better with the aromatase active site than the 2,2-difluorobenzo[*d*][1,3]dioxol-5-yl moiety. However, both **21** (IC_50_=45 nm) and **22** (IC_50_=52 nm) are more potent STS inhibitors, with activities an order of magnitude higher than that of **2** (IC_50_=227 nm). These results suggest that while the 3,5-difluorophenyl ring and the 2,2-difluorobenzo[*d*][1,3]dioxol-5-yl moiety do not improve aromatase inhibition, they confer favourable properties to **21** and **22** for a stronger binding of the compounds to the active site of STS. From this SAR study, it can be concluded that the *para*-cyanophenyl ring provides the best balanced dual inhibition for this class of DASIs.

##### g) Imidazole derivative 25

The most potent DASI studied in this work is compound **25**, the respective IC_50_ values of which against aromatase and STS are 0.2 and 2.5 nm. Compared with **11** (IC_50 arom._=39 nm, IC_50 STS_=21 nm), the replacement of its triazole moiety with an imidazole to give **25** improves both the aromatase and STS inhibitory activities by two orders and one order of magnitude, respectively. (Note: In their patent application, Lafay et al. cited compounds of similar structural features which also possess potent dual inhibition of aromatase and STS.)^38^

The increase in aromatase inhibition observed for **25** is not unexpected, as imidazole (p*K*_BH+_=7.0) is a stronger Lewis base than a triazole (p*K*_BH+_=2.2). The lone-pair electrons on the sp^2^ nitrogen atom of an imidazole are more readily available for ligating to the haem iron centre than the equivalent in a triazole. Therefore, with other structural features being the same, it is anticipated that an imidazole-containing AI will inhibit aromatase more strongly than its triazole-containing counterpart.

**Figure 2:**
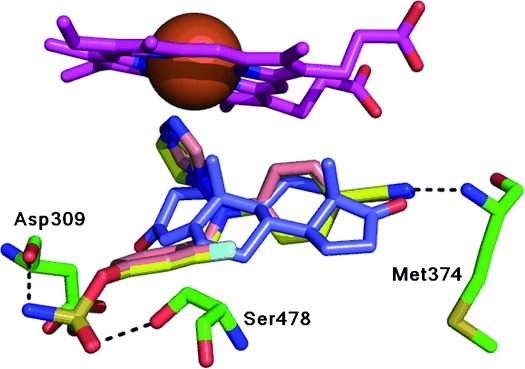
Docking of compounds **11** (pink carbon atoms) and **25** (yellow carbon atoms) into the crystal structure of human aromatase co-crystallised with androstenedione. Selected protein residues are shown with green carbon atoms. The haem is shown with purple carbons, and the iron as a brown sphere. The crystal structure androstenedione has blue carbon atoms. Potential hydrogen bonds are indicated by dashed black lines.

To explore how **25** may interact with the active site of aromatase, we docked the compound and its triazole congener **11** into the crystal structure of human aromatase that had been co-crystallised with androstenedione.^42^, ^43^ Like previous docking studies performed for structurally similar AIs, a distance constraint was set between the haem iron and the ligating nitrogen atom in this work. As shown in Figure 2, the preferred docking poses of **25** (yellow) and **11** (pink) are similar with their heterocycles position under the haem. One small difference observed is that the imidazole group of **25** is 0.1 Å further away from the haem than the triazole group of **11**. This is probably due to steric hindrance posed by the CH group of the imidazole ring of **25** preventing the heterocycle from approaching closer to the iron. The sulfamate group of both **11** and **25** points towards Ser478 and Asp309. Hydrogen bonds (within 2.5–2.6 Å) are anticipated between the sulfamate nitrogen atom and the backbone carbonyl group of Asp309, and also between one of the sulfamate oxygen atoms and the side chain hydroxy group of Ser478. The cyano group of both compounds points towards the same region of space as the C17 carbonyl group of androstenedione, where it may interact with the backbone NH of Met374 as a hydrogen bond acceptor. These docking results of **11** and **25** show the two compounds could potentially bind to active site of aromatase and interact with neighbouring amino acid residues, although the preferred docking poses observed cannot explain the superior aromatase inhibition observed for **25**.

**Figure 3:**
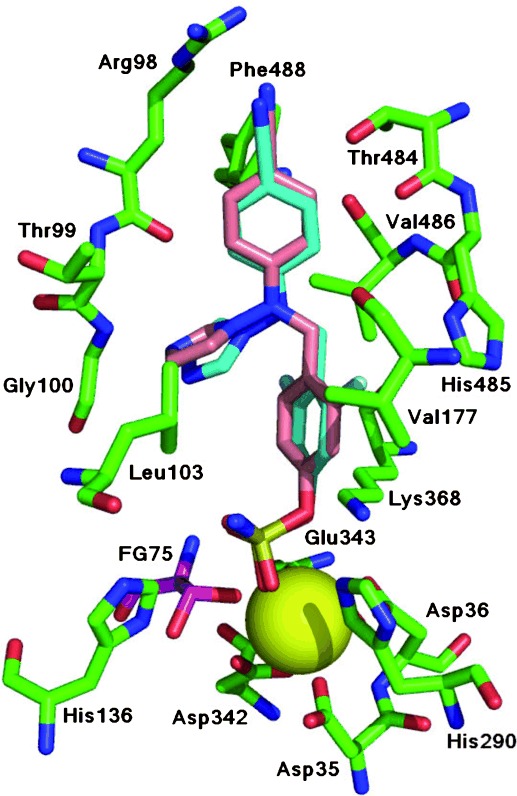
The docking of **11** (cyan) and **25** (pink) into the crystal structure of human STS. Selected protein residues are shown with green carbon atoms. The Ca^2+^ ion is depicted as a yellow sphere, and FG75 (purple) is a *gem*-diol form of formylglycine residue 75.

For STS inhibition, **25** is a potent inhibitor in JEG-3 cells (IC_50_=2.5 nm) with potency similar to that of STS inhibitor **1** (IC_50_=1.5 nm). However, it is not evident why replacing the triazolyl group with an imidazole renders such a significant increase in STS inhibition over that observed for **11**. The docking of **11** and **25** into the crystal structure of human STS^44^ does not reveal any significant difference in their poses. As shown in Figure 3, **11** and **25** bind similarly to STS. Their sulfamate groups interact with FGly75, His290, Lys368, and the calcium ion, whereas their cyano groups point towards the guanidinium group of Arg98. The phenyl ring to which their cyano group is attached may have a face–edge interaction with the ring of Phe488. Despite their different orientations in the docking poses, the imidazole of **25** and the triazole of **11** share the same region of space and may form hydrogen bonds with one or both of Gly100 and Thr99.

Despite the relatively high in vitro potency observed for AIs that contain an imidazole group as the haem-ligating species, it is worth noting that all nonsteroidal AIs currently in clinical use are solely triazole derivatives. Although to the best of our knowledge the preference in featuring a triazole over an imidazole as the haem-ligating species is not clear, one possibility is that triazole compounds are metabolically more stable than their imidazole counterparts in vivo.^45^ It is also possible that triazole-containing aromatase inhibitors are more selective against inhibiting aromatase, whereas imidazole-containing compounds show a wider inhibitory profile against other cytochrome P450 enzymes in addition to aromatase.

#### 2) Parent phenols

We reason that the parent phenols of DASIs can act as AIs in their own right because they possess the minimum pharmacophore for inhibiting aromatase, that is, a haem-ligating moiety such as a triazole or an imidazole. In vivo, at least until all the STS is inhibited, the parent phenol is anticipated to be the immediate product released after irreversible inactivation of STS by an aryl sulfamate according to the various mechanisms of action proposed by our group.^19^, ^46^–^48^ In addition, the parent phenol may also be released if the sulfamate group is hydrolytically cleaved in the plasma due to the limited chemical stability observed for some sulfamates (which increases as the phenolic p*K*_a_ is lowered). Therefore, the parent phenols of all new sulfamates studied in this work were also assessed for their ability to inhibit aromatase.

Previous SAR studies have shown that parent phenols are generally more potent than their corresponding aryl sulfamates as aromatase inhibitors. In this work, however, only **11 c** (*m*-F), **14 h** (*m*-F, *m*′-F, ethylene linker), **15 g** (*o*-F_2_HC), **16 d** (-CF_2_-), **17 c** (*m*-Cl), **19 d** (*m*-Br), and **25 d** (imidazole) follow this general observation, with **12 b** (*m*-F, *m*′-F), **13 c** (*o*-F, *m*-F), **18 c** (*m*-Cl, *m*′-Cl), **20 e** (phenyl), **21 e** (3,5-difluorophenyl), and **22 e** (2,2-difluorobenzo[*d*][1,3]dioxol-5-yl) showing aromatase inhibition either similar to or less potent than their corresponding sulfamates (Table 2).

**Table 2:** In vitro inhibition of aromatase activity in a JEG-3 cell preparation by parent phenols described in this work; compounds 2 a–5 a are included as reference.^23^

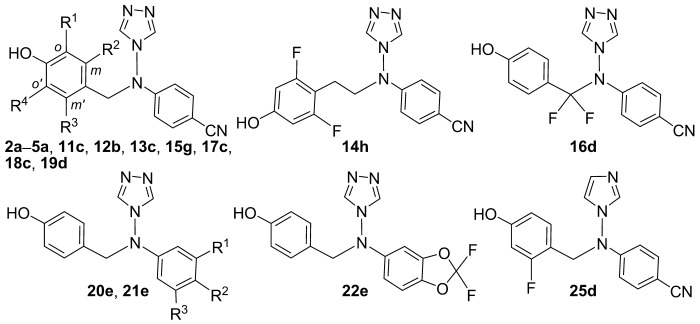					
Compd	R^1^	R^2^	R^3^	R^4^	Aromatase IC_50_ [nm]
**2 a**	H	H	H	H	23
**3 a**	F	H	H	H	2.9
**4 a**	Cl	H	H	H	2.5
**5 a**	Br	H	H	H	1.1
**11 c**	H	F	H	H	3.9±0.6
**12 b**	H	F	F	H	1.3±0.7
**13 c**	F	F	H	H	6.7±1.6
**14 h**	–	–	–	–	0.14±0.03
**15 g**	F_2_HC	H	H	H	1.5±0.3
**16 d**	–	–	–	–	297±25
**17 c**	H	Cl	H	H	3±0.5
**18 c**	H	Cl	Cl	H	2.3±0.2
**19 d**	H	Br	H	H	1.1±0.2
**20 e**	H	H	H	–	3993±12
**21 e**	F	H	F	–	2500±500
**22 e**	–	–	–	–	5500±500
**25 d**	–	–	–	–	0.028±0.004

Relocation of a halogen atom from the *ortho* to the *meta* position to the hydroxy group has little effect on aromatase inhibition, as shown by the similar activities observed for **3 a** (IC_50_=2.9 nm) vs. **11 c** (IC_50_=3.9 nm), **4 a** (IC_50_=2.5 nm) vs. **17 c** (IC_50_=3 nm), and **5 a** (IC_50_=1.1 nm) vs. **19 d** (IC_50_=1.1 nm). In contrast, sulfamates **11**, **17**, and **19** are significantly weaker AIs than **3**, **4**, and **5** respectively.

While adding a second fluoro atom to the remaining *meta* position of **11 c** (IC_50_=3.9 nm) to give the *m*,*m*′-difluorinated compound **12 b** (IC_50_=1.3 nm) improves aromatase inhibition by about threefold, the *m*,*m*′-chlorinated compound **18 c** (IC_50_=2.3 nm) shows similar aromatase inhibitory activity to its *m*-chlorinated counterpart **17 c** (IC_50_=3 nm). These results contrast those of sulfamates **12** and **18**, which are both significantly better AIs than **11** and **17**.

Similar to their sulfamates, the *o*,*m*-difluorinated compound **13 c** (IC_50_=6.7 nm) is a weaker AI than its *m*,*m*′-difluorinated counterpart **12 b** (IC_50_=1.3 nm). This result supports the previous observation that disubstitution of fluorine atoms is more effective for aromatase inhibition if they are substituted at the *m*,*m*′-position rather than at the *o*,*m*-position on the sulfamate-bearing ring.

Replacing the methylene linker of **12 b** (IC_50_=1.3 nm) with an ethylene linker improves the aromatase inhibitory activity of the resulting compound **14 h** (IC_50_=0.14 nm) by nearly tenfold. This observation is not unprecedented, as a similar finding was observed when the methylene linkers of **2 a**–**5 a** were replaced with an ethylene linker.^24^

Replacing the *ortho*-fluorine atom of **3 a** (IC_50_=2.9 nm) with a difluoromethyl group only leads to a moderate increase in potency against aromatase in the resulting compound **15 g** (IC_50_=1.52 nm). In contrast, difluorination of the methylene linker of **2 a** (IC_50_=23 nm) is clearly detrimental to aromatase inhibition, as shown by the ∼13-fold decrease in aromatase inhibitory observed for **16 d** (IC_50_=297 nm).

Like their sulfamate counterparts, the replacement of the *para*-cyanophenyl ring of **2 a** with a phenyl, 3,5-difluorophenyl, or 2,2-difluorobenzo[*d*][1,3]dioxol-5-yl ring is detrimental to aromatase inhibition. The phenols **20 e**, **21 e**, and **22 e** have IC_50_ values two orders of magnitude higher than that of **2 a**.

The most potent in vitro AI in this work is the imidazole derivative **25 d** (IC_50_=0.028 nm) which is about sevenfold more potent than its corresponding sulfamate **25** (IC_50_=0.2 nm) and three orders of magnitude more potent than the triazole counterpart **11**. In addition, the IC_50_ value of **25 d** against aromatase in JEG-3 cells is an order of magnitude higher than that of letrozole (IC_50_=0.89 nm) obtained from the same intact cell assay. However, it is yet to be demonstrated whether this exceptionally high in vitro potency of **25 d** can be reproduced in vivo when the efficacy of a drug is affected by various pharmacokinetic parameters.

## Conclusions

**Figure 4:**
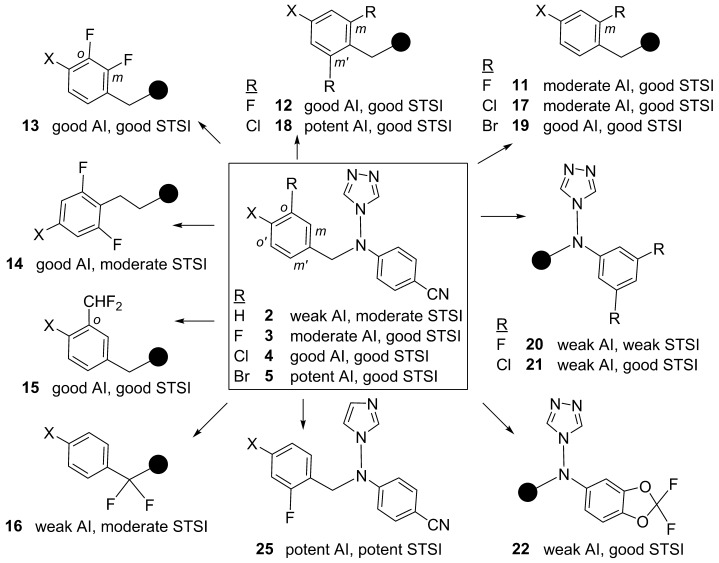
Generalisation of aromatase and STS inhibition exhibited by sulfamates **2**–**5**, **11**–**22**, and **25**. Aromatase inhibition: weak (IC_50_≥100 nm), moderate (10–100 nm), good (1–10 nm), potent (<1 nm); STS inhibition: weak (IC_50_>1000 nm), moderate (100–1000 nm), good (10–100 nm), potent (<10 nm); X=OSO_2_NH_2_; AI: aromatase inhibitor; STSI: STS inhibitor.

Modifications made to first-generation DASIs **2**–**5** produce mixed results. The SAR for the sulfamates is generalised in Figure 4. For STS inhibition, all derivatives show similar STS inhibitory activities unless the *para*-cyanophenyl ring is replaced with a phenyl ring, and the methylene linker is elongated to an ethylene linker or replaced with a difluoromethylene group, where a large decrease in potency is observed. There is a higher degree of variation in the aromatase inhibitory activities observed for the sulfamates, although the most detrimental modifications are replacement of the methylene linker with an ethylene linker, and replacement of the *para*-cyanophenyl ring with a phenyl, 3,5-difluorophenyl, or 2,2-difluorobenzo[*d*][1,3]dioxol-5-yl ring. The most potent DASI discovered in this work is the imidazole derivative **25**, with IC_50_ values against aromatase and STS in JEG-3 cells similar to those of letrozole and **1** (STX64, Figure 1), respectively, obtained from the same assay. The exceptionally potent aromatase inhibition observed for **25** can be attributed to a stronger ligation of its imidazole sp^2^ nitrogen atom to the iron of the haem group than the equivalent in a triazole. Docking of **11** and **25** into the crystal structure of human aromatase shows the two compounds share a similar docking pose. Similarly, docking of the two compounds into the crystal structure of human STS reveals that **11** and **25** share a similar binding mode in the active site.

**Figure 5:**
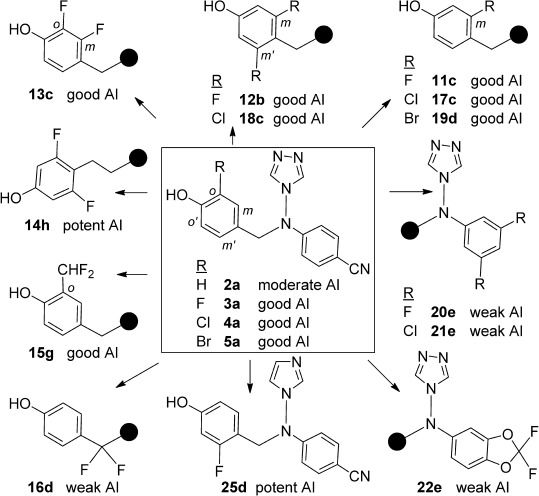
Generalisation of aromatase inhibition exhibited by phenols **2 a**–**5 a**, **11 c**, **12 b**, **13 c**, **14 h**, **15 g**, **17 c**, **18 c**, **19 d**, **20 e**, **21 e**, **22 e**, and **25 d**. Aromatase inhibition: weak (IC_50_≥100 nm), moderate (10–100 nm), good (1–10 nm), potent (<1 nm); AI: aromatase inhibitor

The parent phenols of the sulfamates are good to potent AIs, although replacing the methylene linker with a difluoromethylene group as well as replacing the *para*-cyanophenyl ring with a phenyl, 3,5-difluorophenyl, or 2,2-difluorobenzo[*d*][1,3]dioxol-5-yl ring significantly decreases the aromatase inhibitory activities of the resulting derivatives. As expected, the most potent AI is the imidazole derivative **25 d**. The SAR for the phenols is generalised in Figure 5.

The present work further demonstrates the ability of this structural class of compounds to act as DASIs. Although there is no general breakthrough in designing compounds that exhibit more potent dual inhibition in vitro, interestingly, if the triazole group is replaced by an imidazole as the haem-ligating species, significant activity enhancement is observed both for aromatase and dual aromatase–sulfatase inhibition. The synthesis of more imidazole-containing DASIs is therefore warranted, although to establish real potential for drug design, the in vivo activities of these compounds need to be assessed in parallel in order to evaluate their metabolic stability as well as selectivity towards aromatase inhibition.

## Experimental Section

*In vitro aromatase and sulfatase assays*: Biological activities were performed essentially as described previously.^23^ The extent of in vitro inhibition of aromatase and sulfatase activities was assessed using intact monolayers of JEG-3 human choriocarcinoma cells, which were chosen because these cells constitutively express both enzymes maximally. Aromatase activity was measured with [1β-^3^H]androstenedione (30 Ci mmol^−1^, PerkinElmer Life Sciences, MA, USA) over a 1 h period. Sulfatase activity was measured using [6,7-^3^H]E1S (50 Ci mmol^−1^, PerkinElmer) over a 1 h period.

*Molecular modelling*: The 3EQM crystal structure of aromatase^42^, ^43^ and the 1P49 crystal structure of sulfatase^44^ were used for docking studies. They were passed through the Protein Preparation Wizard of the Schrödinger software platform. GOLD^49^ was used to dock the ligands into both substrate binding sites. [NB: Four new crystal structures of aromatase have recently been released:^50^ 3S79 (with androstenedione) is effectively identical to the 3EQM structure, 3S7S (with exemestane), and 4GL5 and 4GL7, which both have O-linked alkyl chains attached to the 6-position of the steroid ring.]

For aromatase the binding site was centred on the crystal structure ligand (androstenedione) and required the centroid of the docked ligand to be within 5 Å of the androstenedione centroid. A distance constraint of 2.3 Å was applied between the ligating nitrogen atom of the triazole or imidazole ring and the haem iron atom.

For sulfatase the sulfate group was removed from residue formylglycine sulfate 75 (FGS75) to leave a *gem*-diol form of formylglycine residue. The centroid of the docked ligand was required to be within 5 Å of the calcium ion in the substrate binding site. No constraints were applied during the docking.

**General methods for synthesis**: All chemicals were purchased from either Aldrich Chemical Co. (Gillingham, UK) or Alfa Aesar (Heysham, UK). All organic solvents of analytical reagent grade were supplied by Fisher Scientific (Loughborough, UK). Anhydrous *N*,*N*-dimethylformamide (DMF), *N*,*N*-dimethylacetamide (DMA) and tetrahydrofuran (THF) were purchased from Aldrich. Sulfamoyl chloride was prepared by an adaptation of the method of Appel and Berger^51^ and was stored as a solution under N_2_ in toluene as described by Woo et al.^46^

Thin-layer chromatography (TLC) was performed on pre-coated aluminium plates (Merck, silica gel 60 F_254_). Product spots were visualised either by UV irradiation at *λ* 254 nm or by staining with either an alkaline solution of KMnO_4_ or 5 % *w*/*v* dodecamolybdophosphoric acid in EtOH, followed by heating. Flash column chromatography was performed on silica gel (Davisil silica 60A) or pre-packed columns (Isolute), and gradient elution (solvents indicated in text) on either the Flashmaster II system (Biotage) or on a Teledyne ISCO CombiFlash *R*_f_ Automated Flash Chromatography System with RediSep *R*_f_ disposable flash columns. ^1^H and ^13^C NMR spectra were recorded with either a Jeol Delta 270 MHz or a Varian Mercury VX 400 MHz spectrometer. Chemical shifts (*δ*) are reported in parts per million (ppm) relative to tetramethylsilane (TMS) as an internal standard. Coupling constants (*J*) are recorded to the nearest 0.1 Hz. Mass spectra were recorded at the Mass Spectrometry Service Centre, University of Bath (UK). FAB mass spectra were carried out with *m*-nitrobenzyl alcohol as the matrix. Elemental analyses were performed by the Microanalysis Service, University of Bath. Melting points were determined with either a Stuart Scientific SMP3 or a Stanford Research Systems Optimelt MPA100 and are uncorrected.

**LC–MS and HPLC**: LC–MS was performed using a Waters 2790 instrument with a ZQ MicroMass spectrometer and PDA detector. The ionisation technique used was either APCI or ES (as indicated in the text). A Waters *Symmetry* C_18_ (packing: 3.5 μm) 4.6×100 mm column with gradient elution 5:95 CH_3_CN/H_2_O (flow rate: 0.5 mL min^−1^) to 95:5 CH_3_CN/H_2_O (flow rate: 1 mL min^−1^) over 10 min were used. HPLC was undertaken using a Waters 717 machine with Autosampler and PDA detector. The column used was a Waters *Symmetry* C_18_ (packing: 3.5 μm) 4.6×150 mm with an isocratic mobile phase consisting of MeOH/H_2_O (as indicated) at a flow rate of 1.4 mL min^−1^.

**General method A—hydrogenation**: Pd/C was added to a solution of the substrate in the solvents indicated. The solution was stirred under an atmosphere of H_2_ (provided by addition from a balloon) overnight. The excess H_2_ was removed, and the reaction mixture was filtered through Celite washing with THF and MeOH, then the solvent was removed in vacuo.

**General method B—sulfamoylation**: A solution of sulfamoyl chloride (H_2_NSO_2_Cl) in toluene was concentrated in vacuo at 30 °C to furnish a yellow oil which solidified upon cooling in an ice bath. DMA and the substrate were subsequently added, and the mixture was allowed to warm to room temperature and stirred overnight. The reaction mixture was poured onto H_2_O and extracted three times with EtOAc. The organic layers were combined, washed four times with H_2_O, and then with brine, dried (MgSO_4_), and the solvent was removed in vacuo.

**Methyl 2-fluoro-4-hydroxybenzoate (11 a)**: A solution of 2-fluoro-4-hydroxybenzoic acid (5.30 g, 34.0 mmol) and conc. HCl (30 drops) in MeOH (100 mL) was heated at reflux for 12 h. The mixture was allowed to cool and was neutralised with sat. aq. NaHCO_3_. The solvent was removed in vacuo, and the residue was dissolved in EtOAc (100 mL) and washed with H_2_O (100 mL), sat. aq. NaHCO_3_ (100 mL), and brine (100 mL), then dried (MgSO_4_), and the solvent was removed in vacuo. The title compound was obtained as a white powder (4.52 g, 78 %): mp: 154–156 °C; ^1^H NMR (270 MHz, [D_6_]DMSO): *δ*=3.79 (3 H, s, C*H*_3_), 6.63 (1 H, dd, *J*=13.2, 2.2 Hz, Ar*H*), 6.70 (1 H, dd, *J*=8.5, 2.2 Hz, Ar*H*), 7.77 (1 H, t, *J*=8.8 Hz, Ar*H*), 10.83 ppm (1 H, s, O*H*).

**3-Fluoro-4-(hydroxymethyl)phenol (11 b)**: A solution of **11 a** (1.50 g, 8.82 mmol) in THF (10 mL) was added to a suspension of LiAlH_4_ (0.84 g, 22.1 mmol) in THF (35 mL). After 45 min the reaction was quenched by cautious addition of EtOAc (15 mL), H_2_O (15 mL), and 3 m HCl (15 mL). The product was extracted with EtOAc (2×75 mL), and the combined organic layers were washed with sat. aq. NaHCO_3_ (100 mL), dried (MgSO_4_), and the solvent was removed in vacuo to give the title compound as a cream solid (0.95 g, 76 %): mp: 111–113 °C; ^1^H NMR (270 MHz, [D_6_]DMSO): *δ*=4.40 (2 H, d, *J*=5.2 Hz, C*H*_2_), 5.02 (1 H, t, *J*=5.2 Hz, O*H*), 6.51 (1 H, dd, *J*=11.9, 2.5 Hz, Ar*H*), 6.58 (1 H, dd, *J*=8.3, 2.5 Hz, Ar*H*), 7.21 (1 H, t, *J*=8.8 Hz, ArH), 9.76 ppm (1 H, br s, O*H*).

**4-[(2-Fluoro-4-hydroxybenzyl)(4*H*-1,2,4-triazol-4-yl)amino]benzonitrile (11 c)**: A solution of **11 b** (0.82 g, 4.43 mmol) in SOCl_2_ (5 mL) was stirred for 2 h, then the SOCl_2_ was removed in vacuo. The residue was dissolved in DMF (8 mL), and **10** (0.60 g, 4.23 mmol) and K_2_CO_3_ (2.92 g, 21.2 mmol) were added, and the reaction mixture was stirred for 48 h. The mixture was poured onto H_2_O (30 mL), and the product was extracted with EtOAc (2×30 mL). The combined organic layers were washed with H_2_O (4×30 mL) and brine (30 mL), then dried (MgSO_4_), and the solvent was removed in vacuo. The crude product was purified using the CombiFlash system (CHCl_3_/acetone) to give the title compound as a white powder (0.26 g, 20 %): mp: 217–219 °C; ^1^H NMR (270 MHz, [D_6_]DMSO): *δ*=4.96 (2 H, s, C*H*_2_), 6.49–6.55 (2 H, m, Ar*H*), 6.76 (2 H, AA′BB′, Ar*H*), 7.04 (1 H, d, *J*=8.2 Hz, Ar*H*), 7.76 (2 H, AA′BB′, Ar*H*), 8.71 (2 H, s, 2×C*H*N), 10.09 ppm (1 H, s, O*H*); ^13^C NMR (100 MHz, [D_6_]DMSO): *δ*=50.7 (CH_2_), 102.5 (C), 102.7 (CH), 111.0 (C, d), 111.7 (CH, d), 113.6 (2×CH), 119.1 (C), 132.1 (CH, d), 133.9 (2×CH), 143.4 (2×CH), 151.3 (C), 159.3 (C, d), 161.3 ppm (C, d); ^19^F NMR (376 MHz, [D_6_]DMSO): *δ*=−116.1 ppm t; LC–MS (ES+): *t*_R_=1.37 min, *m*/*z* (%): 310.0 (100) [*M*+H]^+^; HRMS (ES+): *m*/*z* [*M*+H]^+^ calcd for C_16_H_13_FN_5_O: 310.1099, found: 310.1099; HPLC: *t*_R_=1.29 min (>99 %), (CH_3_CN/H_2_O, 90:10).

**4-{[(4-Cyanophenyl)(4*H*-1,2,4-triazol-4-yl)amino]methyl}-3-fluorophenyl sulfamate (11)**: As general method B using ClSO_2_NH_2_ (0.63 m, 4.4 mL), DMA (2.5 mL) and **11 c** (0.17 g, 0.55 mmol). The crude product was purified using the CombiFlash system (CHCl_3_/acetone) to give the title compound as a white amorphous solid (0.17 g, 80 %): ^1^H NMR (270 MHz, [D_6_]DMSO): *δ*=5.15 (2 H, s, C*H*_2_), 6.75 (2 H, AA′BB′, Ar*H*), 7.08 (1 H, dd, *J*=8.6, 2.2 Hz, Ar*H*), 7.17 (1 H, dd, *J*=10.7, 2.2 Hz, Ar*H*), 7.46 (1 H, t, *J*=8.6 Hz, Ar*H*), 7.77 (2 H, AA′BB′, Ar*H*), 8.17 (2 H, s, N*H*_2_), 8.83 ppm (2 H, s, 2×C*H*N); ^13^C NMR (100 MHz, [D_6_]DMSO): *δ*=50.9 (CH_2_), 103.0 (C), 109.9 (CH, d), 113.6 (2×CH), 118.3 (CH), 118.6 (C, d), 120.0 (C, d), 131.6 (CH, d), 134.0 (2×CH), 143.3 (2×CH), 150.9 (C, d), 151.1 (C), 159.9 ppm (C, d); ^19^F NMR (376 MHz, [D_6_]DMSO): *δ*=−114.1 ppm t; LC–MS (ES+): *t*_R_=1.14 min, *m*/*z* (%): 389.0 (100) [*M*+H]^+^; HRMS (ES+): *m*/*z* [*M*+H]^+^ calcd for C_16_H_14_FN_6_O_3_S: 389.0827, found: 389.0819; HPLC: *t*_R_=1.26 min (>99 %), (CH_3_CN/H_2_O, 90:10).

**3,5-Difluoro-4-(hydroxymethyl)phenol (12 a)**: 2,6-Difluoro-4-hydroxybenzoic acid (0.50 g, 2.87 mmol) was cautiously added to a suspension of LiAlH_4_ (0.55 g, 14.5 mmol) in THF (20 mL). The resulting mixture was stirred at reflux for 24 h. After cooling to room temperature, the reaction was quenched by the cautious addition of EtOAc (10 mL) and then H_2_O (10 mL). A further portion of EtOAc (50 mL) was added and the layers were separated. The aqueous layer was extracted with EtOAc (50 mL) and the combined organic layers were washed with 3 m aq. HCl (2×50 mL), then dried (MgSO_4_) and the solvent was removed in vacuo to give title compound as a white solid (0.40 g, 87 %): ^1^H NMR (270 MHz, [D_6_]DMSO): *δ*=4.36 (2 H, d, *J*=5.4 Hz, C*H*_2_), 5.03 (1 H, d, *J*=5.4 Hz, O*H*), 6.39–6.50 (2 H, m, Ar*H*), 10.43 ppm (1 H, s, OH); LC–MS (ES^−^): *t*_R_=0.90 min, *m*/*z* (%): 158.9 (100) [*M*−H]^−^, 140.9 (60).

**4-[(2,6-Difluoro-4-hydroxybenzyl)(4*H*-1,2,4-triazol-4-yl)amino]benzonitrile (12 b)**: A solution of **12 a** (0.37 g, 2.31 mmol) in SOCl_2_ (5 mL) was stirred for 2 h, then the SOCl_2_ was removed in vacuo. The residue was dissolved in DMF (7 mL) and **10** (0.45 g, 2.43 mmol) and K_2_CO_3_ (1.60 g, 11.6 mmol) were added and the reaction mixture was stirred overnight. The mixture was poured onto H_2_O (30 mL) and extracted with EtOAc (2×30 mL). The combined organics were washed with H_2_O (4×30 mL) and brine (30 mL), then dried (MgSO_4_) and the solvent was removed in vacuo. The crude product was purified using Flashmaster II (CH_2_Cl_2_/acetone) to give the title compound as a white solid (0.23 g, 30 %); mp: 217 °C (dec.); ^1^H NMR (270 MHz, [D_6_]DMSO): *δ*=4.99 (2 H, s, C*H*_2_), 6.38–6.48 (2 H, m, Ar*H*), 6.79 (2 H, m, AA′BB′, ArH), 7.77 (2 H, m, AA′BB′, ArH), 8.73 (2 H, s, 2×C*H*N), 10.49 ppm (1 H, s, OH); ^13^C NMR (100 MHz, [D_6_]DMSO): *δ*=44.6 (CH_2_), 99.4 (2×CH, d), 100.2 (C, t), 102.9 (C), 113.6 (2×CH), 119.1 (C), 134.0 (2×CH), 143.4 (2×CH), 151.0 (C), 160.0 (C, t), 161.8 ppm (2×C, dd); ^19^F NMR (376 MHz, [D_6_]DMSO): *δ*=−114.7 ppm d; LC–MS (ES+): *t*_R_=3.11 min, *m*/*z* (%): 328.2 (100) [*M*+H]^+^, 259.1 (40); HRMS (ES+): *m*/*z* [*M*+H]^+^ calcd for C_16_H_12_F_2_N_5_O: 328.1004, found: 328.1008; HPLC: *t*_R_=1.43 min (98.3 %), (CH_3_CN/H_2_O, 90:10).

**4-{[(4-Cyanophenyl)(4*H*-1,2,4-triazol-4-yl)amino]methyl}-3,5-difluorophenyl sulfamate (12)**: As general method B using ClSO_2_NH_2_ (0.6 m, 5.0 mL), DMA (3 mL) and **12 b** (0.15 g, 0.46 mmol). The crude product was purified using Flashmaster II (CH_2_Cl_2_/acetone) to give the title compound (0.14 g, 75 %) as a white amorphous solid; ^1^H NMR (270 MHz, [D_6_]DMSO): *δ*=5.16 (2 H, s, C*H*_2_), 6.81 (2 H, m, AA′BB′, Ar*H*), 7.09 (2 H, m, Ar*H*), 7.80 (2 H, AA′BB′, Ar*H*), 8.33 (2 H, s, N*H*_2_), 8.80 ppm (2 H, s, 2×C*H*N); ^13^C NMR (600 MHz, [D_6_]DMSO): *δ*=44.8 (CH_2_), 103.2 (C), 106.3 (2×CH, d), 108.8 (C, t), 113.7 (2×CH), 119.0 (C), 134.1 (2×CH), 143.4 (2×CH), 150.9 (C), 151.4 (C, d), 161.2 ppm (2×C, m); ^19^F NMR (376 MHz, [D_6_]DMSO): *δ*=−112.3 ppm s; LC–MS (ES+): *t*_R_=1.06 min, *m*/*z* (%): 405.0 (100) [*M*−H]^−^; HRMS (ES+): *m*/*z* [*M*+H]^+^ calcd for C_16_H_13_F_2_N_6_O_3_S: 407.0732, found: 407.0726; HPLC: *t*_R_=1.42 min (>99 %), (CH_3_CN/H_2_O, 90:10).

**Methyl 2,3-difluoro-4-hydroxybenzoate (13 a)**: SOCl_2_ (0.63 g, 4.57 mmol) was added to a solution of 2,3-difluoro-4-hydroxybenzoic acid (0.20 g, 1.15 mmol) in MeOH (10 mL) and the mixture was heated at reflux for 24 h. After cooling to room temperature the solvent was removed in vacuo to give the title compound as a beige powder in a quantitative yield: mp: 138–140 °C; ^1^H NMR (270 MHz, [D_6_]DMSO): *δ*=3.81 (3 H, s, C*H*_3_), 6.87 (1 H, td, *J*=9.1, 2.2 Hz, Ar*H*), 7.57 (1 H, td, *J*=9.1, 2.2 Hz, Ar*H*), 11.41 ppm (1 H, br s, O*H*); LC–MS (ES^−^): *t*_R_=0.85 min, *m*/*z* (%): 186.7 (100) [*M*−H]^−^.

**2,3-Difluoro-4-(hydroxymethyl)phenol (13 b)**: A solution of **13 a** (0.17 g, 0.90 mmol) in THF (2 mL) was added to a suspension of LiAlH_4_ (0.10 g, 2.63 mmol) in THF (5 mL). After 45 min the reaction was quenched by the cautious addition of EtOAc (3 mL), H_2_O (3 mL) and 3 m HCl (3 mL). The product was extracted with EtOAc (2×15 mL) and the combined organics were washed with sat. aq. NaHCO_3_ (20 mL) dried (MgSO_4_) and the solvent was removed in vacuo to give the title compound as an off-white solid (0.14 g, 94 %): mp: 114 °C (dec.); ^1^H NMR (270 MHz, [D_6_]DMSO): *δ*=4.42 (2 H, d, *J*=5.3 Hz, C*H*_2_), 5.17 (1 H, t, *J*=5.3 Hz, O*H*), 6.74 (1 H, td, *J*=8.3, 2.2 Hz, Ar*H*), 7.00 (1 H, td, *J*=8.3, 2.2 Hz, Ar*H*), 10.26 ppm (1 H, br s, OH); ^13^C NMR (100 MHz, [D_6_]DMSO): *δ*=56.5 (CH_2_), 112.4 (CH), 120.8 (C), 123.3 (CH), 139.5 (C), 145.5 (C), 148.6 ppm (C); LC–MS (ES^−^): *t*_R_=0.82 min, *m*/*z* (%): 158.8 (100) [*M*−H]^−^; HRMS (ES+): *m*/*z* [*M*+Na]^+^ calcd for C_7_H_6_F_2_NaO_2_:183.0228, found: 183.0220.

**4-[(2,3-Difluoro-4-hydroxybenzyl)(4*H*-1,2,4-triazol-4-yl)amino]benzonitrile (13 c)**: A solution of **13 b** (0.24 g, 1.50 mmol) in SOCl_2_ (5 mL) was stirred for 2 h, then the SOCl_2_ was removed in vacuo. The residue was dissolved in DMF (5 mL) and **10** (0.29 g, 1.58 mmol) and K_2_CO_3_ (1.04 g, 7.53 mmol) were added and reaction mixture stirred for 72 h. The mixture was poured onto H_2_O (50 mL) and extracted with EtOAc (2×30 mL). The combined organics were washed with H_2_O (4×30 mL) and brine (30 mL), then dried (MgSO_4_), and the solvent was removed in vacuo. The crude product was purified using Flashmaster II (EtOAc/hexane) and (CH_2_Cl_2_/acetone) to give the title compound as a white solid (0.15 g, 15 %): mp: 225 °C (dec.); ^1^H NMR (270 MHz, [D_6_]DMSO): *δ*=5.02 (2 H, s, C*H*_2_), 6.6.3–6.89 (4 H, m, Ar*H*), 7.77 (2 H, m, AA′BB′, ArH), 8.76 (2 H, s, 2×C*H*N), 10.58 ppm (1 H, s, O*H*); ^13^C NMR (100 MHz, [D_6_]DMSO): *δ*=50.7 (CH_2_), 102.9 (C), 112.7 (CH), 112.8 (2×CH), 119.0 (C), 125.2 (C, CH), 133.9 (2×CH), 139.5 (C, dd), 143.4 (2×CH), 147.1 (2×C, m), 149.5 (C, dd), 151.1 ppm (C); ^19^F NMR (376 MHz, [D_6_]DMSO): *δ*=−141.4 (dd), −161.1 ppm (dd); LC–MS (ES+): *t*_R_=0.76 min, *m*/*z* (%): 350.0 (100) [*M*+Na]^+^, 328 (20), 258.9 (15); HRMS (ES+): *m*/*z* [*M*+H]^+^ calcd for C_16_H_12_F_2_N_5_O: 328.1004, found: 328.0990; HPLC *t*_R_=1.23 min (>99 %), (CH_3_CN/H_2_O, 90:10).

**4-{[(4-Cyanophenyl)(4*H*-1,2,4-triazol-4-yl)amino]methyl}-2,3-difluorophenyl sulfamate (13)**: As general method B using ClSO_2_NH_2_ (0.6 m, 3.4 mL), DMA (3 mL) and **13 c** (0.11 g, 0.34 mmol). The crude product was purified using Flashmaster II (CH_2_Cl_2_/acetone) to give the title compound as an amorphous white solid (0.14 g, 95 %); ^1^H NMR (270 MHz, [D_6_]DMSO): *δ*=5.21 (2 H, s, C*H*_2_), 6.75 (2 H, m, AA′BB′, Ar*H*), 7.18–7.31 (2 H, m, Ar*H*), 7.77 (2 H, AA′BB′, Ar*H*), 8.41 (2 H, s, N*H*_2_), 8.88 ppm (2 H, s, 2×C*H*N); ^13^C NMR (100 MHz, [D_6_]DMSO): *δ*=55.9 (CH_2_), 103.1 (C), 113.6 (2×CH), 119.0 (CH), 119.7 (C), 122.5 (C, d), 124.7 (CH), 134.1 (2×CH), 138.5 (C, m), 143.2 (C, dd), 143.4 (2×CH), 148.9 (C, dd), 150.9 ppm (C); ^19^F NMR (376 MHz, [D_6_]DMSO): *δ*=−151.5 (dd), −138.6 ppm (dd); LC–MS (ES+): *t*_R_=0.87 min, *m*/*z* (%): 407.0 (100) [*M*+H]^+^; HRMS (ES+): *m*/*z* [*M*+H]^+^ calcd for C_16_H_13_F_2_N_6_O_3_S: 407.0732, found: 407.0722; HPLC: *t*_R_=1.34 min (>99 %), (CH_3_CN/H_2_O, 90:10).

**2-(2,6-Difluoro-4-methoxyphenyl)malonic acid (14 a)**: 3 m aq. NaOH (80 mL) added to a solution of diethyl 2-(2,6-difluoro-4-methoxyphenyl)malonate (synthesised according to a patented procedure;^34^ 6.30 g, 20.85 mmol) in Et_2_O (5 mL). The reaction mixture was heated at reflux until all the solid had dissolved. After cooling, the mixture was acidified with 3 m aq. HCl and the product was extracted with EtOAc (2×100 mL). The combined organic layers were dried (MgSO_4_) and solvent was removed in vacuo to give the title compound as a tan-coloured solid (4.50 g, 88 %): mp: 157 °C (dec.); ^1^H NMR (270 MHz, [D_6_]DMSO): *δ*=3.78 (3 H, s, C*H*_3_), 4.72 (1 H, s, C*H*), 6.70–6.79 ppm (2 H, m, Ar*H*).

**2-(2,6-Difluoro-4-methoxyphenyl)acetic acid (14 b)**: **14 a** (4.49 g, 18.3 mmol) was heated at 160 °C until melting was complete and fizzing had ceased. Following cooling, the product was recrystallised from petroleum ether (PE)/EtOAC to give the title compound as a white crystalline solid (3.43 g, 93 %): mp: 137–139 °C; ^1^H NMR (270 MHz, [D_6_]DMSO): *δ*=3.52 (2 H, s, C*H*_2_), 3.77 (3 H, s, C*H*_3_), 6.68–6.79 ppm (2 H, m, Ar*H*); HRMS (ES+): *m*/*z* [*M*+H]^+^ calcd for C_9_H_8_F_2_O_3_: 203.0514, found: 203.0508.

**Methyl 2-(2,6-difluoro-4-methoxyphenyl)acetate (14 c)**: A solution of **14 b** (3.37 g, 16.7 mmol) in MeOH (70 mL) containing conc. HCl (15 drops) was heated at reflux overnight. The mixture was allowed to cool and was neutralised with sat. aq. NaHCO_3_. The solvent was removed in vacuo and the residue was dissolved in EtOAc (100 mL) and washed with H_2_O (100 mL), sat. aq. NaHCO_3_ (100 mL) and brine (100 mL) then dried (MgSO_4_) and the solvent was removed in vacuo. The title compound was obtained as a pale-yellow oil (3.30 g, 92 %); ^1^H NMR (270 MHz, [D_6_]DMSO): *δ*=3.61 (2 H, s, C*H*_2_), 3.69 (3 H, s, C*H*_3_), 3.76 (3 H, s, C*H*_3_), 6.40–6.49 ppm (2 H, m, Ar*H*); ^19^F NMR (376.4 MHz, [D_6_]DMSO): *δ*=−114.4 ppm (d); LC–MS (ES+): *t*_R_=1.69 min, *m*/*z* (%): 216.8 (100) [*M*]^+^; HRMS (ES+): *m*/*z* [*M*+H]^+^ calcd for C_10_H_11_F_2_O_3_: 217.0657, found: 217.0663.

**Methyl 2-(2,6-difluoro-4-hydroxyphenyl)acetate (14 d)**: BBr_3_ (1 m in CH_2_Cl_2_, 55 mL, 55 mmol) was added to a cooled (−78 °C) solution of **14 c** (2.87 g, 13.3 mmol) in CH_2_Cl_2_ (25 mL). The reaction mixture was stirred at −78 °C for 10 min, then allowed to warm to room temperature and stirred for 3.5 h. The reaction was quenched with sat. aq. NaHCO_3_ and the product was extracted with CH_2_Cl_2_ (2×150 mL). The combined organic layers were washed with brine (150 mL), dried (MgSO_4_) and the solvent was removed in vacuo. The crude product was purified using the CombiFlash system (PE/EtOAc) to give the title compound as a white crystalline solid (2.07 g, 77 %): mp: 75.5–77.5 °C; ^1^H NMR (270 MHz, [D_6_]DMSO): *δ*=3.58 (2 H, s, C*H*_2_), 3.61 (3 H, s, C*H*_3_), 6.41–6.51 (2 H, m, Ar*H*), 10.34 ppm (1 H, s, O*H*); ^19^F NMR (376.4 MHz, [D_6_]DMSO): *δ*=−115.4 ppm (d); LC–MS (ES+): *t*_R_=1.45 min, *m*/*z* (%): 202.8 (100) [*M*+H]^+^; HRMS (ES+): *m*/*z* [*M*+H]^+^ calcd for C_9_H_9_F_2_O_3_: 203.0514, found: 203.0511.

**Methyl 2-[2,6-difluoro-4-(triisopropylsilyloxy)phenyl]acetate (14 e)**: Imidazole (1.43 g, 21.0 mmol) and triisopropylsilyl chloride (TIPSCl, 2.24 g, 11.6 mmol) were sequentially added to a pale-yellow solution of **14 d** (2.13 g, 10.5 mmol) in DMF (10 mL). The reaction mixture was stirred overnight, then poured onto H_2_O (40 mL) and extracted with EtOAc (3×50 mL). The combined organics were washed with H_2_O (3×100 mL) and brine (100 mL), then dried (MgSO_4_) and the solvent was removed in vacuo. The crude product was purified using the CombiFlash system (PE/EtOAc) to give the title compound as a colourless oil (3.43 g, 91 %); ^1^H NMR (270 MHz, CDCl_3_): *δ*=1.04–1.32 (21 H, m, 6×C*H*_3_, 3×C*H*), 3.60 (2 H, s, C*H*_2_), 3.69 (3 H, s, C*H*_3_), 6.36–6.48 ppm (2 H, m, Ar*H*); ^13^C NMR (100 MHz, [D_6_]DMSO): *δ*=12.5 (3×CH), 17.8 (6×CH_3_), 27.4 (CH_2_), 52.2 (CH_3_), 103.3 (C, t), 103.4 (2×CH, dd), 156.7 (C, t), 161.6 (2×C, dd), 170.6 ppm (C); ^19^F NMR (376 MHz, [D_6_]DMSO): *δ*=−117.9 ppm (d).

**2-[2,6-Difluoro-4-(triisopropylsilyloxy)phenyl]ethanol (14 f)**: A solution of **14 e** (2.98 g, 8.30 mmol) in THF (15 mL) was added to a cooled (0 °C) suspension of LiAlH_4_ (0.79 g, 20.8 mol) in THF (50 mL). After stirring for 15 min, the reaction was quenched by the sequential addition of EtOAc (25 mL), H_2_O (25 mL) and 3 m HCl (25 mL). The product was extracted with EtOAc (2×75 mL) and the combined organic layers were washed with sat. aq. NaHCO_3_ (2×100 mL) dried (MgSO_4_) and the solvent was removed in vacuo. The crude product was purified using the CombiFlash system (EtOAc/PE) to give the title compound as a colourless oil (1.70 g, 62 %); ^1^H NMR (270 MHz, CDCl_3_): *δ*=1.02–1.31 (21 H, m, 6×CH_3,_ 3×CH_),_ 1.61 (1 H, s, O*H*), 2.85 (2 H, t, *J*=6.9 Hz, C*H*_2_), 3.76 (2 H, t, *J*=6.9 Hz, C*H*_2_), 6.34–6.48 ppm (2 H, m, Ar*H*); ^19^F NMR (376 MHz, [D_6_]DMSO): *δ*=−116.0 ppm (d); LC–MS (ES+): *t*_R_=2.23 min, *m*/*z* (%): 353.4 (100) [*M*+Na]^+^, 331.3 (80) [*M*+1]^+^; HRMS (ES+): *m*/*z* [*M*+H]^+^ calcd for C_17_H_29_F_2_O_2_Si: 331.1899, found: 331.1887.

**[4-(2-Bromoethyl)-3,5-difluorophenoxy]triisopropylsilane (14 g)**: Triphenylphosphine (PPh_3_, 1.11 g, 4.24 mmol) was added to a cooled (0 °C) solution of CBr_4_ (0.92 g, 2.77 mmol) in CH_2_Cl_2_ (15 mL). After stirring for 15 min, a solution of **14 f** (0.70 g, 2.12 mmol) in CH_2_Cl_2_ (5 mL) was added and the reaction mixture was stirred for 1 h, then allowed to warm to room temperature and stirred for 2 h. The solvent was removed in vacuo and the residue was purified using the CombiFlash system (EtOAc/PE) to give the title compound as a colourless oil (0.56 g, 67 %); ^1^H NMR (270 MHz, CDCl_3_): *δ*=1.05–1.32 (21 H, m, 6×CH_3,_ 3×CH)_,_ 3.13 (2 H, t, *J*=7.7 Hz, C*H*_2_), 3.48 (2 H, t, *J*=7.7 Hz, C*H*_2_), 6.34–6.45 ppm (2 H, m, Ar*H*).

**4-[(2,6-Difluoro-4-hydroxyphenethyl)(4*H*-1,2,4-triazol-4-yl)amino]benzonitrile (14 h)**: NaH (60 % dispersion in mineral oil, 0.057 g, 1.42 mmol) was added to a solution of **14 g** (0.24 g, 1.30 mmol) in DMF (7 mL). After stirring for 30 min a solution of **10** (0.56 g, 1.42 mmol) in DMF (3 mL) was added and stirring was continued for 48 h. TBAF (1 m in THF, 1.75 mL) was added and after 30 min the reaction mixture was poured onto H_2_O (15 mL) and acidified with 3 m aq. AcOH. The product was extracted with EtOAc (3×20 mL) and the combined organics were washed with H_2_O (4×50 mL) and brine (50 mL) then dried (MgSO_4_) and the solvent was removed in vacuo. The crude product was purified using the CombiFlash system (CHCl_3_/acetone) to give the title compound as a white solid (0.18 g, 41 %): mp: 275 °C (dec.); ^1^H NMR (270 MHz, [D_6_]DMSO): *δ*=2.79 (2 H, t, *J*=7.5 Hz, C*H*_2_), 3.96 (2 H, t, *J*=7.5 Hz, C*H*_2_), 6.41–6.50 (2 H, m, Ar*H*), 6.58 (2 H, AA′BB′, Ar*H*), 7.73 (2 H, AA′BB′, Ar*H*), 8.87 (2 H, s, 2×C*H*N), 10.32 ppm (1 H, s, O*H*); ^13^C NMR (100 MHz, [D_6_]DMSO): *δ*=19.4 (CH_2_), 52.7 (CH_2_), 99.2 (2×CH, d), 102.4 (C), 102.6 (C), 112.8 (2×CH), 119.0 (C), 134.0 (2×CH), 143.4 (2×CH), 150.6 (C), 158.1 (C, t), 161.3 ppm (2×C, dd); ^19^F NMR (376 MHz, [D_6_]DMSO): *δ*=−116.0 ppm (d); LC–MS (ES+): *t*_R_=1.26 min, *m*/*z* (%): 342.2 (100) [*M*+H]^+^; HRMS (ES+): *m*/*z* [*M*+H]^+^ calcd for C_17_H_14_F_2_N_5_O: 342.1161, found: 342.1153; HPLC: *t*_R_=1.31 min (98.5 %), (CH_3_CN/H_2_O, 90:10).

**4-{2-[(4-Cyanophenyl)(4*H*-1,2,4-triazol-4-yl)amino]ethyl}-3,5-difluorophenyl sulfamate (14)**: As general method B using ClSO_2_NH_2_ (0.45 m, 4.0 mL), DMA (2 mL) and **14 h** (0.095 g, 0.28 mmol). The crude product was purified using the CombiFlash system (CHCl_3_/acetone) to give the title compound (0.062 g, 53 %) as an amorphous solid; ^1^H NMR (270 MHz, [D_6_]DMSO): *δ*=2.92 (2 H, t, *J*=7.5 Hz, C*H*_2_), 4.04 (2 H, t, *J*=7.5 Hz, C*H*_2_), 6.60 (2 H, AA′BB′, Ar*H*), 7.06–7.13 (2 H, m, Ar*H*), 7.75 (2 H, AA′BB′, Ar*H*), 8.25 (2 H, br s, N*H*_2_), 8.93 ppm (2 H, s, 2×C*H*N); LC–MS (ES+): *t*_R_=1.19 min, *m*/*z* (%): 421.1 (100) [*M*+H]^+^; HRMS (ES+): *m*/*z* [*M*+H]^+^ calcd for C_17_H_15_F_2_N_6_O_3_S: 421.0889, found: 421.0895; HPLC: *t*_R_=1.26 min (>99 %), (CH_3_CN/H_2_O, 90:10).

**3-Formyl-4-hydroxybenzoic acid methyl ester (15 a)**: This compound was prepared according to the method described by Hofsløkken and Skattebol;^35^ yield=38 %.

**3-Difluoromethyl-4-hydroxybenzoic acid methyl ester (15 b)**: A solution of **15 a** (1.40 g, 7.78 mmol) in CH_2_Cl_2_ (20 mL) was added to a solution of Deoxo-Fluor® (2.92 g, 13.2 mmol) in CH_2_Cl_2_ then EtOH (0.072 g, 89 μL, 1.56 mol) was added and the resultant was stirred for 6 h. The reaction mixture was quenched by the cautious addition of sat. aq. NaHCO_3_ then CH_2_Cl_2_ (75 mL was added). The layers were separated and the organic washed with H_2_O (75 mL) and brine (75 mL), then dried (MgSO_4_) and the solvent was removed in vacuo. The crude product was purified using Flashmaster II (EtOAc/hexane) to give the title compound as a white solid (1.18 g, 75 %): mp: 141–142 °C; ^1^H NMR (270 MHz, [D_6_]DMSO): *δ*=3.91 (3 H, s, C*H*_3_), 6.56 (1 H, br s, O*H*), 6.88 (1 H, t, *J*=55.2 Hz, C*H*F_2_), 6.93 (1 H, d, *J*=8.6 Hz, Ar*H*), 8.02 (1 H, d, *J*=8.6 Hz, Ar*H*), 8.16 ppm (1 H, s, Ar*H*); ^13^C NMR (100 MHz, [D_6_]DMSO): *δ*=52.4 (CH_3_), 112.4 (CH, t), 116.7 (CH), 120.8 (C), 120.9 (C, t), 128.1 (CH, t), 134.0 (CH), 160.4 (C, t), 165.9 ppm (C); LC–MS (APCI^−^): *t*_R_=2.74 min, *m*/*z* (%): 200.9 (100) [*M*−H]^−^, 180.9 (75), 152.8 (50), 138.8 (20); HRMS (ES+): *m*/*z* [*M*+H]^+^ calcd for C_9_H_9_F_2_O_3_: 203.0514, found: 203.0514.

**3-Difluoromethyl-4-triisopropylsilanyloxybenzoic acid methyl ester (15 c)**: Imidazole (0.79 g, 11.6 mmol) and TIPSCl (1.24 g, 6.42 mmol) were sequentially added to a solution of **15 b** (1.18 g, 5.84 mmol) in DMF (15 mL). The reaction mixture was stirred overnight, then poured onto H_2_O (50 mL) and extracted with EtOAc (3×50 mL). The combined organics were washed with H_2_O (3×75 mL) and brine (75 mL), then dried (MgSO_4_) and the solvent was removed in vacuo. The crude product was purified using Flashmaster II (EtOAc/hexane) to give the title compound as a colourless oil (2.11 g, 99 %): ^1^H NMR (400 MHz, CDCl_3_): *δ*=1.06–1.42 (21 H, m, 3×C*H*, 6×C*H*_3_), 3.89 (3 H, s, OC*H*_3_), 6.86 (1 H, d, *J*=8.6 Hz, Ar*H*), 6.91 (1 H, t, *J*=55.0 Hz, C*H*_2_F), 8.00 (1 H, d, *J*=8.6 Hz, Ar*H*), 8.23 ppm (1 H, s, ArH); ^13^C NMR (100 MHz, [D_6_]DMSO): *δ*=12.94 (3×CH), 17.64 (6×CH_3_), 52.1 (CH_3_), 111.4 (CH, t), 118.4 (CH), 122.9 (C), 124.7 (C, t), 128.7 (CH, t), 133.4 (CH), 158.2 (C, t), 166.2 ppm (C); LC–MS (APCI): *t*_R_=1.75 min, *m*/*z* (%) 359.3 (100) [*M*+H]^+^; HRMS (ES+): *m*/*z* [*M*+H]^+^ calcd for C_18_H_29_F_2_O_3_Si: 359.1849, found: 359.1846.

**(3-Difluoromethyl-4-triisopropylsilanyloxyphenyl)methanol (15 d)**: LiBH_4_ (0.31 g, 14.1 mmol) and B(OMe)_3_ (0.058 g, 0.56 mmol) were sequentially added to a solution of **15 c** (2.00 g, 5.58 mmol) in Et_2_O (70 mL). The reaction mixture was stirred overnight and was then diluted with Et_2_O (70 mL) and quenched by the cautious addition of H_2_O (70 mL). The layers were separated and the organic layer was washed with brine (70 mL), then dried (MgSO_4_) and the solvent was removed in vacuo. The crude product was purified using the Flashmaster II system (EtOAc/hexane) to give the title compound as a colourless oil (1.23 g, 67 %): ^1^H NMR (300 MHz, CDCl_3_): *δ*=1.02–1.31 (21 H, m, 3×C*H*, 6×C*H*_3_), 1.56 (1 H, t, *J*=5.1 Hz, O*H*), 4.57 (2 H, d, *J*=5.1 Hz, C*H*_2_), 6.77 (1 H, d, *J*=8.3 Hz, Ar*H*), 6.88 (1 H, t, *J*=55.0 Hz, C*H*_2_F), 7.25 (1 H, d, *J*=8.3 Hz, Ar*H*), 7.46 ppm (1 H, s, Ar*H*); ^13^C NMR (68 MHz, CDCl_3_): *δ*=13.0 (3×CH), 18.0 (6×CH_3_), 64.9 (CH_2_), 111.9 (CH, t), 118.6 (CH), 124.5 (C, t), 125.5 (CH, t), 130.7 (CH), 133.4 (C), 153.8 ppm (C, t); LRMS (FAB^+^): *m*/*z* (%): 331.4 (10) [*M*]^+^, 313.1 (100); HRMS (ES+): *m*/*z* [*M*+Na]^+^ calcd for C_17_H_28_F_2_NaO_2_SiBr: 353.1719, found: 353.1719.

**(4-Bromomethyl-2-difluoromethylphenoxy)triisopropylsilane (15 e)**: A solution of PBr_3_ (0.26 g, 0.96 mmol) in CH_2_Cl_2_ (6 mL) was added dropwise to a cooled (0 °C) solution of **15 d** (0.64 g, 1.94 mmol) in CH_2_Cl_2_ (6 mL). The reaction mixture was allowed to warm to room temperature and was stirred for 1 h. Aqueous NaHCO_3_ (sat., 10 mL) and CH_2_Cl_2_ (30 mL) were added and the layers were separated. The organic layer was washed with H_2_O (2×50 mL) and brine (50 mL) then dried (MgSO_4_) and the solvent was removed in vacuo. The solvent was removed in vacuo, and the crude product was purified using Flashmaster II (EtOAc/hexane) to give the title compound as a white crystalline solid (0.58 g, 76 %): mp: 43–45 °C; ^1^H NMR (270 MHz, CDCl_3_): *δ*=1.02–1.31 (21 H, m, 3×C*H*, 6×C*H*_3_), 4.41 (2 H, s, C*H*_2_), 6.74 (1 H, d, *J*=8.5 Hz, Ar*H*), 6.85 (1 H, t, *J*=55.7 Hz, C*H*_2_F), 7.28 (1 H, d, *J*=8.5 Hz, Ar*H*), 7.49 ppm (1 H, d, *J*=2.1 Hz, Ar*H*); ^13^C NMR (68 MHz, CDCl_3_): *δ*=13.0 (3×CH), 18.0 (6×CH_3_), 32.2 (CH_2_), 111.6 (CH, t), 118.8 (CH), 124.9 (C, t), 127.4 (CH, t), 130.3 (C), 132.6 (CH), 154.3 ppm (C, t); LRMS (FAB^+^): *m*/*z* (%): 393.1 (100) [*M*]^+^, 313.1 (80), 227.1 (50); HRMS (ES+): *m*/*z* [*M*+Na]^+^ calcd for C_17_H_27_F_2_NaOSiBr: 415.0875, found: 415.0875.

**4-[(3-Difluoromethyl-4-triisopropylsilanyloxybenzyl)-[1,2,4]triazol-4-ylamino]benzonitrile (15 f)**: **15 e** (0.18 g, 0.98 mmol) was added to a cooled (0 °C) suspension of NaH (60 % in mineral oil, 0.039 g, 0.98 mmol) in DMF (12 mL) and the resulting solution was stirred for 30 min at room temperature. A solution of **10** (0.42 g, 1.07 mmol) in DMF (3 mL) was added and after stirring overnight the reaction mixture was poured onto H_2_O (40 mL) and the product was extracted with EtOAc (2×40 mL). The combined organics were washed with H_2_O (4×60 mL) and brine (60 mL), then dried (MgSO_4_) and the solvent was removed in vacuo. The crude product was purified using Flashmaster II (EtOAc/hexane) to give the title compound as a white solid (0.084 g, 74 %): mp: 85.0–87.5 °C; ^1^H NMR (270 MHz, CDCl_3_): *δ*=1.03–1.35 (21 H, m, 3×C*H*, 6×C*H*_3_), 4.83 (2 H, s, C*H*_2_), 6.66 (2 H, AA′BB′, Ar*H*), 6.79 (1 H, d, *J*=8.4, Ar*H*), 6.90 (1 H, t, *J*=55.4 Hz, C*H*F_2_), 7.06 (1 H, d, *J*=8.4 Hz, Ar*H*), 7.42 (1 H, s, Ar*H*), 7.58 (2 H, AA′BB′, Ar*H*), 8.09 ppm (2 H, s, 2×C*H*N); ^13^C NMR (68 MHz, CDCl_3_): *δ*=12.9 (3×CH), 18.0 (6×CH_3_), 57.7 (CH_2_), 105.4 (C), 111.3 (CH, t), 113.6 (2×CH), 118.6 (CH), 119.4 (CH), 125.6 (C), 125.7 (C, t), 126.5 (CH, t), 131.6 (CH), 134.3 (2×CH), 142.7 (2×CH), 150.5 (C), 154.9 ppm (C, t); LCMS (APCI^+^): *m*/*z* (%): 498.5 (100) [*M*+H]^+^, 429.5 (15); HRMS (ES+): *m*/*z* [*M*+H]^+^ calcd for C_26_H_34_F_2_N_5_OSi: 498.2495, found: 498.2463.

**4-{[3-(Difluoromethyl)-4-hydroxybenzyl)(4*H*-1,2,4-triazol-4-yl]amino}benzonitrile (15 g)**: A solution of TBAF (IM in THF, 0.5 mL) was added to a solution of **15 f** (0.32 g, 0.64 mmol) in THF (6 mL) and the reaction mixture was stirred for 15 min. A few drops of AcOH were added until the solution became colourless and then the reaction mixture was poured onto H_2_O (20 mL) and the product extracted with EtOAc (2×20 mL). The combined organic layers were washed with H_2_O (40 mL), sat. NaHCO_3_ solution (40 mL) and brine (40 mL), then dried (MgSO_4_) and the solvent was removed in vacuo. The crude product was purified by trituration with hexane to give the title compound as a white solid (0.19 g, 95 %): mp: 175 °C (dec.); ^1^H NMR (270 MHz, [D_6_]DMSO): *δ*=4.99 (2 H, s, C*H*_2_), 6.75–6.86 (3 H, m, Ar*H*), 6.99 (1 H, t, *J*=55.4 Hz, C*H*F_2_), 7.22 (1 H, d, *J*=8.4 Hz, Ar*H*), 7.33 (1 H, s, Ar*H*), 7.77 (2 H, AA′BB′, Ar*H*), 8.74 (2 H, s, 2×C*H*N), 10.35 ppm (1 H, s, O*H*); ^13^C NMR (68 MHz, [D_6_]DMSO): *δ*=56.8 (CH_2_), 103.4 (C), 112.7 (CH, t), 114.4 (2×CH), 116.7 (CH), 119.6 (C), 120.8 (C, t), 125.4 (C), 127.2 (CH, t), 133.1 (CH), 134.5 (2×CH), 143.9 (2×CH), 152.1 (C), 156.0 ppm (C, t); ^19^F NMR (376 MHz, CDCl_3_): *δ*=−115.2 ppm (d); LCMS (APCI^+^): *m*/*z* (%) 340.3 (100) [*M*−H]^−^, 271.3 (20); HRMS (FAB^+^): *m*/*z* [*M*+H]^+^ calcd for C_17_H_14_F_2_N_5_O: 342.1161, found: 342.1158; HPLC: *t*_R_=1.75 min (95 %), (CH_3_CN/H_2_O, 90:10).

**4-{[(4-Cyanophenyl)(4*H*-1,2,4-triazol-4-yl)amino]methyl}-2-(difluoromethyl)phenyl sulfamate (15)**: As general method B using ClSO_2_NH_2_ (0.6 m, 2.2 mL), DMA (2 mL) and **15 g** (0.090 g, 0.26 mmol). The title compound was obtained as a white solid (0.10 g, 90 %): mp: 148–151 °C; ^1^H NMR (270 MHz, [D_6_]DMSO): *δ*=5.19 (2 H, s, C*H*_2_), 6.76 (2 H, m, AA′BB′), 7.10 (1 H, t, *J*=55.4 Hz, C*H*F_2_), 7.43 (1 H, d, *J*=8.4 Hz, Ar*H*), 7.58–7.67 (2 H, m, Ar*H*), 7.78 (2 H, m, AA′BB′), 8.38 (2 H, s, N*H*_2_), 8.87 ppm (2 H, s, 2×C*H*N); ^13^C NMR (68 MHz, [D_6_]DMSO): *δ*=56.9 (CH_2_), 103.6 (C), 111.7 (CH, t), 114.3 (2×CH), 119.6 (C), 123.4 (CH), 127.0 (CH, t), 127.5 (C) 127.9 (C), 132.8 (CH), 134.5 (2×CH), 143.9 (2×CH), 148.0 (C, t), 151.9 ppm (C); LCMS (APCI^−^): *m*/*z* (%): 419.3 (100) [*M*−H]^−^, 340.4 (20), 271.3 (20); HRMS (FAB^+^): *m*/*z* [*M*+H]^+^ calcd for C_17_H_15_N_6_O_3_F_2_S: 421.0889, found: 421.0889; HPLC: *t*_R_=1.81 min (>99 %), (CH_3_CN/H_2_O, 90:10).

**4-Benzyloxy-*N*-(4-cyanophenyl)-*N*-[1,2,4]triazol-4-ylbenzamide (16 a)**: A suspension of 4-benzyloxybenzoic acid (1.00 g, 4.39 mmol) in SOCl_2_ (10 mL) was heated at reflux until gas evolution ceased and was then allowed to cool and SOCl_2_ was removed in vacuo. The residue was suspended in CH_2_Cl_2_ (40 mL) and **10** (0.65 g, 3.51 mmol) and Et_3_N (3.55 g, 35.1 mmol) were sequentially added and the mixture was stirred for 2 h. The reaction mixture was concentrated in vacuo and the residue was dissolved in EtOAc (60 mL) and washed with 1 m aq. HCl (60 mL), H_2_O (60 mL), NaHCO_3_ (60 mL) and brine (60 mL). The organic layer was dried (MgSO_4_) and the solvent was removed in vacuo. The crude product was purified using Flashmaster II (EtOAc/hexane) to give the title compound as a white crystalline solid (0.92 g, 67 %): mp: 205–207.5 °C; ^1^H NMR (270 MHz, [D_6_]DMSO): *δ*=5.09 (2 H, s, C*H*_2_), 7.00 (2 H, s, AA′BB′), 7.27–7.53 (9 H, m, Ar*H*), 7.88 (2 H, s, AA′BB′, Ar*H*), 9.10 ppm (2 H, s, 2×C*H*N); ^13^C NMR (100 MHz, [D_6_]DMSO): *δ*=70.0 (CH_2_), 110.8 (C), 115.1 (2×CH), 118.5 (C), 124.3 (C), 126.7 (2×CH), 128.5 (2×CH), 128.6 (CH), 129.0 (2×CH), 131.5 (2×CH), 134.3 (2×CH), 136.8 (C), 143.9 (2×CH), 145.7 (C), 161.5 (C), 167.8 ppm (C); LC–MS (APCI^−^): *t*_R_=3.38 min, *m*/*z* (%): 396.3 (100) [*M*+H]^+^, 327.3 (60), 299.3 (20); HRMS (ES+): *m*/*z* [*M*+H]^+^ calcd for C_23_H_18_N_5_O_2_: 396.1455, found: 396.1444.

**4-(Benzyloxy)-*N*-(4-cyanophenyl)-*N*-(4*H*-1,2,4-triazol-4-yl)benzothioamide (16 b)**: A mixture of **16 a** (1.40 g, 3.54 mmol) and Lawesson′s reagent (1.07 g, 2.65 mmol) in xylenes (140 mL) was heated at reflux for 42 h. The reaction mixture was allowed to cool and the solvent was removed in vacuo. The residue was dissolved in EtOAc (75 mL) and washed with H_2_O (75 mL) and brine (75 mL) then dried (MgSO_4_) and the solvent was removed in vacuo. The crude product was purified using Flashmaster II (EtOAc/hexane) to give the title compound as a yellow solid (0.81 g, 56 %): ^1^H NMR (270 MHz, CDCl_3_) *δ*=5.10 (2 H, s, C*H*_2_), 6.94 (2 H, AA′BB′, Ar*H*), 7.32–7.45 (5 H, m, Ar*H*), 7.50–7.59 (4 H, m, Ar*H*), 7.89 (2 H, AA′BB′, Ar*H*), 9.19 ppm (2 H, s, 2×C*H*N); LC–MS (APCI^+^): *t*_R_=4.41 min, *m*/*z* (%): 412.4 (100) [*M*+H]^+^, 214.3 (20), 119.1 (30). HRMS (FAB+): *m*/*z* [*M*+H]^+^ calcd for C_23_H_18_N_5_OS: 412.1227, found 412.1230.

**4-{[(4-(Benzyloxy)phenyl)difluoromethyl](4*H*-1,2,4-triazol-4-yl)amino}benzonitrile (16 c)**: SbCl_3_ (0.007 g, 0.031 mmol) and Deoxo-Fluor® (0.17 g, 0.77 mmol) were sequentially added to a solution of **16 b** (0.12 g, 0.29 mmol) in CH_2_Cl_2_ (3 mL). The reaction mixture was stirred overnight and was quenched by the cautious addition of sat. aq. NaHCO_3_ (20 mL). CH_2_Cl_2_ (15 mL) was added and the layers were separated and the organic layer was washed with H_2_O (15 mL) then dried (MgSO_4_) and the solvent was removed in vacuo. The crude product was purified using Flashmaster II (EtOAc/hexane) to give the title compound as a yellow solid (0.087 g, 71 %): mp: 167–169 °C; ^1^H NMR (270 MHz, [D_6_]DMSO): *δ*=5.11 (2 H, s, C*H*_2_), 7.09 (2 H, AA′BB′, Ar*H*), 7.32–7.48 (7 H, m, Ar*H*), 7.66 (2 H, AA′BB′, Ar*H*), 7.87 (2 H, AA′BB′, Ar*H*), 9.11 ppm (2 H, s, 2×C*H*N); ^13^C NMR (68 MHz, [D_6_]DMSO): *δ*=70.1 (CH_2_), 110.0 (C), 115.6 (2×CH), 118.6 (C), 121.9 (t, C), 123.3 (t, C), 124.5 (2×CH), 128.5 (2×CH), 128.6 (CH), 129.0 (2×CH), 129.0 (2×CH), 134.5 (2×CH), 136.9 (C), 144.5 (2×CH), 145.1 (C), 161.3 ppm (C); LC–MS (APCI^+^): *t*_R_=4.49 min, *m*/*z* (%): 418.3 (100) [*M*+H]^+^; HRMS (FAB+): *m*/*z* [*M*+H]^+^ calcd for C_23_H_18_F_2_N_5_O: 418.1474, found: 418.1474.

**4-{[Difluoro(4-hydroxyphenyl)methyl](4*H*-1,2,4-triazol-4-yl)amino}benzonitrile (16 d)**: As general method A using 10 % Pd/C (8 mg), **16 c** (0.04 g, 0.096 mmol) and THF/MeOH (1:1, 4 mL). The crude product was purified using Flashmaster II (EtOAc/hexane) to give the title compound as a white solid (0.019 g, 61 %): mp: 143–145 °C; ^1^H NMR (270 MHz, [D_6_]DMSO): *δ*=6.79 (2 H, AA′BB′, Ar*H*), 7.36 (2 H, AA′BB′, Ar*H*), 7.50 (2 H, AA′BB′, Ar*H*), 7.87 (2 H, AA′BB′, Ar*H*), 9.08 (2 H, s, 2×C*H*N), 10.21 ppm (1 H, br s, O*H*); ^13^C NMR (100 MHz, [D_6_]DMSO): *δ*=109.3 (C), 115.6 (2×CH), 118.1 (C), 120.9 (t, C), 121.6 (t, C), 123.7 (2×CH), 128.4 (2×CH), 133.9 (2×CH), 144.0 (2×CH), 144.7 (C), 160.2 ppm (C); LC–MS (APCI^+^): *t*_R_=1.25 min, *m*/*z* (%): 327.46 (80) [*M*]^+^, 21.3.1 (100); HRMS (FAB+): *m*/*z* [*M*+H]^+^ calcd for C_16_H_12_F_2_N_5_O: 328.1004, found: 328.1004; HPLC: *t*_R_=3.54 min (>99 %), (CH_3_CN/H_2_O, 70:30).

**4-[*N*-(4-cyanophenyl)-*N*-(4*H*-1,2,4-triazol-4-yl)fluorocarbonyl]phenyl sulfamate (16)**: As general method B using ClSO_2_NH_2_ (0.6 m, 1.2 mL), DMA (3 mL) and **16 d** (0.050 g, 0.15 mmol). The crude product was purified by precipitation from EtOAc/hexane to give the title compound as a white solid (0.045 g, 76 %): mp: 139.5–142 °C; ^1^H NMR (270 MHz, [D_6_]DMSO): *δ*=7.38 (2 H, AA′BB′, Ar*H*), 7.44 (2 H, AA′BB′, Ar*H*), 7.88 (4 H, 2×AA′BB′, Ar*H*), 8.21 (2 H, s, SO_2_N*H*_2_), 9.14 ppm (2 H, s, 2×C*H*N); ^13^C NMR (100 MHz, [D_6_]DMSO): *δ*=109.9 (C), 118.0 (C), 120.7 (t, C), 122.3 (t, C), 122.5 (2×CH), 124.3 (2×CH), 128.7 (2×CH), 134.1 (2×CH), 143.9 (2×CH), 144.2 (C), 152.6 ppm (C); LC–MS (APCI^−^): *t*_R_=2.88 min, *m*/*z* (%): 405.4 (100) [*M*−H]^−^, 212.0 (60), 184 (50); HRMS (FAB^+^): *m*/*z* [*M*+H]^+^ calcd for C_16_H_13_F_2_N_6_O_3_S: 407.0732, found: 407.0734; HPLC: *t*_R_=10.73 min (97.3 %), (CH_3_CN/H_2_O, 50:50).

**3-Chloro-4-(hydroxymethyl)phenol (17 a)**: This was prepared according to the method described by Matysiak et al.;^52^ yield=14 %.

**3-Chloro-4-(chloromethyl)phenol (17 b)**: A solution of **17 a** (0.10 g, 0.63 mmol) in SOCl_2_ (2 mL) was stirred for 2 h. The reaction mixture was concentrated in vacuo and the product was used directly in next step: ^1^H NMR (270 MHz, [D_6_]DMSO): *δ*=4.74 (2 H, s, C*H*_2_), 6.75 (1 H, dd, *J*=8.4 Hz, 2.5 Ar*H*), 6.88 (1 H, d, *J*=2.5 Hz, Ar*H*), 7.39 ppm (1 H, d, *J*=8.4 Hz, Ar*H*).

**4-[(2-Chloro-4-hydroxybenzyl)(4*H*-1,2,4-triazol-4-yl)amino]benzonitrile (17 c)**: Compound **10** (0.12 g, 0.64 mmol) and K_2_CO_3_ (0.43 g, 3.12 mmol) were sequentially added to a solution of **17 b** in DMF (5 mL). The reaction mixture was stirred overnight and then poured onto H_2_O (25 mL) and extracted with EtOAc (2×25 mL). The organic layers were combined, washed with H_2_O (3×25 mL) and brine (25 mL), dried (MgSO_4_) and the solvent was removed in vacuo. The crude product was purified using Flashmaster II (EtOAc/hexane) to give the title compound as a white powder (0.18 g, 100 %): ^1^H NMR (270 MHz, [D_6_]DMSO): *δ*=5.00 (2 H, s, C*H*_2_), 6.62 (1 H, dd, *J*=8.4 Hz, 2.5, Ar*H*), 6.78–6.80 (3 H, m, Ar*H*), 7.05 (1 H, d, *J*=8.4 Hz, Ar*H*), 7.76 (2 H, AA′BB′, Ar*H*), 8.71 (2 H, s, 2×C*H*N), 10.05 ppm (1 H, s, OH); ^13^C NMR (100 MHz, [D_6_]DMSO): *δ*=54.7 (CH_2_), 103.0 (C), 113.6 (2×CH), 119.0 (C), 121.3 (CH), 123.2 (CH), 130.5 (C), 131.6 (CH), 133.5 (C), 134.1 (2×CH), 143.4 (2×CH), 150.3 (C), 151.2 ppm (C); LC–MS (APCI^+^): *t*_R_=0.95 min, *m*/*z* (%): 326.4 (3) [*M*+H]^+^, 257.3 (100), 141.1 (40); HRMS (FAB+): *m*/*z* [*M*+H]^+^ calcd for C_16_H_13_ClN_5_O: 326.0803, found: 326.0804; HPLC: *t*_R_=3.14 min (99 %), (CH_3_CN/H_2_O, 90:10).

**3-Chloro-4-{[(4-cyanophenyl)(4*H*-1,2,4-triazol-4-yl)amino]methyl}phenyl sulfamate (17)**: As general method B using ClSO_2_NH_2_ (0.6 m, 3 mL), DMA (3 mL) and **17 c** (0.12 g, 0.37 mmol). The crude product was purified using Flashmaster II (EtOAc/hexane) and was then precipitated from EtOAc/hexane to give the title compound as a white amorphous solid (0.12 g, 81 %): ^1^H NMR (270 MHz, [D_6_]DMSO): *δ*=5.18 (2 H, s, C*H*_2_), 6.72 (2 H, AA′BB′, Ar*H*), 7.20 (1 H, dd, *J*=8.4 Hz, 2.2, Ar*H*), 7.42 (1 H, d, *J*=2.2 Hz, Ar*H*), 7.48 (1 H, d, *J*=8.4 Hz, Ar*H*), 7.77 (2 H, AA′BB′, Ar*H*), 8.19 (2 H, s, N*H*_2_), 8.85 ppm (2 H, s, 2×C*H*N); ^13^C NMR (100 MHz, [D_6_]DMSO): *δ*=54.7 (CH_2_), 103.0 (C), 113.6 (2×CH), 119.0 (C), 121.3 (CH), 123.2 (CH), 130.5 (C), 131.6 (CH), 133.5 (C), 134.1 (2×CH), 143.4 (2×CH), 150.3 (C), 151.2 ppm (C); LC–MS (ES^−^): *t*_R_=0.91 min, *m*/*z* (%): 403.4 (100) [*M*−H]^−^; HRMS (ES^−^): *m*/*z* [*M*−H]^−^ calcd for C_16_H_12_ClN_6_O_3_S: 403.0386, found: 403.0386; HPLC: *t*_R_=3.26 min (99 %), (CH_3_CN/H_2_O, 90:10).

**2,6-Dichloro-4-hydroxybenzaldehyde (18 a)**: This was prepared according to the method as described by Bringmann et al.^53^

**3,5-Dichloro-4-(hydroxymethyl)phenol (18 b)**: A solution of NaBH_4_ (0.070 g, 1.84 mmol) in H_2_O (0.5 mL) was added dropwise to a solution of **18 a** (0.13 g, 0.68 mmol) in EtOH (2 mL). After stirring for 1 h, the reaction was quenched by the cautious addition of aq. HCl (3 m, 5 mL), EtOAc (10 mL) and H_2_O (10 mL). The layers were separated and the aqueous was extracted with EtOAc (10 mL). The organic layers were combined, washed with H_2_O (2×20 mL) and aq. sat. NaHCO_3_ (20 mL) then dried (MgSO_4_) and the solvent was removed in vacuo to give the title compound as a white crystalline solid (0.12 g, 93 %): mp: 169–171 °C; ^1^H NMR (270 MHz, [D_6_]DMSO): *δ*=4.56 (2 H, d, *J*=5.0 Hz, C*H*_2_), 5.01 (1 H, t, *J*=5.0 Hz, Ar*H*), 6.82 (2 H, s, Ar*H*), 10.40 ppm (1 H, s, O*H*); LC–MS (ES+): *m*/*z* (%): 191.1 (100) [*M*]^+^.

**4-[(2,6-Dichloro-4-hydroxybenzyl)(4*H*-1,2,4-triazol-4-yl)amino]benzonitrile (18 c)**: A solution of **18 b** (0.30 g, 1.56 mmol) in SOCl_2_ (3 mL) was stirred for 2 h (with heating when needed to dissolve) then the SOCl_2_ was removed in vacuo. The residue was dissolved in DMF (6 mL) and **10** (1.01 g, 5.46 mmol) and K_2_CO_3_ (3.60 g, 26.1 mmol) were added and the reaction mixture was stirred for 48 h. The mixture was poured onto H_2_O (50 mL) and the product was extracted with EtOAc (2×30 mL). The combined organic layers were washed with H_2_O (4×30 mL) and brine (30 mL), then dried (MgSO_4_) and the solvent was removed in vacuo. The crude product was purified using the CombiFlash system (CH_2_Cl_2_/acetone) to give the title compound as a white solid (0.71 g, 38 %): mp: 230 °C (dec.); ^1^H NMR (270 MHz, [D_6_]DMSO): *δ*=5.17 (2 H, s, C*H*_2_), 6.78.3–6.86 (4 H, m, Ar*H*), 7.77 (2 H, m, AA′BB′, ArH), 8.63 (2 H, s, 2×C*H*N), 10.60 ppm (1 H, s, O*H*); ^13^C NMR (100 MHz, [D_6_]DMSO): *δ*=50.7 (CH_2_), 102.7 (C), 113.5 (2×CH), 115.8 (2×CH), 119.1 (C), 119.4 (C), 133.9 (2×CH), 136.4 (2×C) 143.5 (2×CH), 151.0 (C), 158.6 ppm (C); LC–MS (ES+): *t*_R_=1.32 min, *m*/*z* (%): 360.2 (100) [*M*]^+^; HRMS (ES+): *m*/*z* [*M*]^+^ calcd for C_16_H_12_Cl_2_N_5_O: 360.0413, found: 360.0405; HPLC: *t*_R_=1.35 min (>99 %), (CH_3_CN/H_2_O, 90:10).

**3,5-Dichloro-4-{[(4-cyanophenyl)(4*H*-1,2,4-triazol-4-yl)amino]methyl}phenyl sulfamate (18)**: As general method B using ClSO_2_NH_2_ (0.5 m, 8.0 mL), DMA (3 mL) and **18 d** (0.20 g, 0.56 mmol). The crude product was purified using Flashmaster II (CH_2_Cl_2_/acetone) to give the title compound as a white amorphous solid (0.13 g, 55 %): ^1^H NMR (270 MHz, [D_6_]DMSO): *δ*=5.32 (2 H, s, C*H*_2_), 6.84 (2 H, m, AA′BB′, Ar*H*), 7.42 (2 H, s, Ar*H*), 7.79 (2 H, m, AA′BB′, Ar*H*), 8.35 (2 H, br s, N*H*_2_), 8.69 ppm (2 H, s, 2×C*H*N); ^13^C NMR (100 MHz, [D_6_]DMSO): *δ*=50.9 (CH_2_), 103.1 (C), 113.7 (2×CH), 119.0 (C), 122.3 (2×CH), 126.1 (C), 134.0 (2×CH), 136.5 (2×C), 143.5 (2×CH), 150.4 (C), 150.9 ppm (C); LC–MS (ES+): *t*_R_=1.30 min, *m*/*z* (%): 439.0 (100) [*M*+H]^+^; HRMS (ES+): *m*/*z* [*M*+H]^+^ calcd for C_16_H_13_Cl_2_N_6_OS: 439.0141, found: 439.0141; HPLC: *t*_R_=1.31 min (>99 %), (CH_3_CN/H_2_O, 90:10).

**3-Bromo-4-methylphenyl benzoate (19 a)**: Benzoyl chloride (1.65 g, 11.7 mmol) was added dropwise to a solution of 3-bromo-4-methylphenol (2.00 g, 10.7 mmol) and pyridine (3 mL) in CH_2_Cl_2_ (20 mL). The solution was stirred for 1 h at room temperature, then H_2_O was added and stirring was continued for 0.5 h. The organic layer was separated, washed with 2 m KHSO_4_ (20 mL) and brine (10 mL), dried (MgSO_4_) and concentrated in vacuo. The residue was recrystallised from MeOH to afford the title compound as a white solid (2.35 g, 75 %): mp: 74–75 °C (lit.^54^ mp: 75–76 °C); ^1^H NMR (400 MHz, [D_6_]DMSO): *δ*=2.42 (3 H, s, C*H*_3_), 7.09 (1 H, dd, *J*=8.6 Hz, 2.3 Hz, Ar*H*), 7.29 (1 H, d, *J*=8.6 Hz, Ar*H*), 7.44 (1 H, d, *J*=2.3 Hz, Ar*H*), 7.48–7.54 (2 H, m, Ar*H*), 7.61–7.68 (1 H, m, Ar*H*), 8.16–8.22 ppm (2 H, m, Ar*H*); ^13^C NMR (100 MHz, [D_6_]DMSO): *δ*=22.3 (CH_3_), 120.6 (CH), 124.5 (C), 125.6 (CH), 128.6 (2×CH), 129.2 (C), 130.2 (2×CH), 131.0 (CH), 133.7 (CH), 135.5 (C), 149.1 (C), 164.9 ppm (C); LRMS (ES+): *m*/*z* (%): 290.6 (100) [*M*+H]^+^.

**3-Bromo-4-(bromomethyl)phenyl benzoate (19 b)**: A mixture of **19 a** (2.30 g, 7.90 mmol), NBS (1.79 g, 7.90 mmol) and benzoyl peroxide (5 mg) in CCl_4_ (20 mL) was heated at reflux for 1 h 30 min with small additional portions of benzoyl peroxide added every 15 min. After cooling to room temperature, the reaction mixture was filtered and the solvent was removed in vacuo from the filtrate. The crude product was purified using the CombiFlash system (PE/EtOAc) to give the title compound as a white solid (1.49 g, 51 %): ^1^H NMR (400 MHz, [D_6_]DMSO): *δ*=4.62 (2 H, s, CH_2_), 7.21 (1 H, dd, *J*=8.4, 2.4 Hz, Ar*H*), 7.50–7.54 (4 H, m, Ar*H*), 7.63–7.68 (1 H, m, Ar*H*), 8.16–8.19 ppm (2 H, m, Ar*H*); ^13^C NMR (100 MHz, [D_6_]DMSO): *δ*=32.5 (CH_2_), 121.5 (CH), 124.5 (C), 126.7 (CH), 128.7 (2×CH), 129.0 (C), 130.3 (2×CH), 131.8 (CH), 134.0 (CH), 134.7 (C), 151.2 (C), 1645.6 ppm (C).

**3-Bromo-4-{[(4-cyanophenyl)(4*H*-1,2,4-triazol-4-yl)amino]methyl}phenyl benzoate (19 c)**: NaH (60 % dispersion in mineral oil, 0.048 g, 1.20 mmol) was added to a solution of **10** (0.21 g, 1.14 mmol) in DMF (10 mL). After stirring for 30 min, **19 b** (0.38 g, 1.03 mmol) was added and stirring was continued for 48 h. The reaction mixture was poured onto H_2_O (25 mL) and the product was extracted with EtOAc (3×30 mL). The combined organic layers were washed with H_2_O (4×60 mL) and brine (60 mL) then dried (MgSO_4_) and the solvent was removed in vacuo. The crude product was purified using the CombiFlash system (CH_2_Cl_2_/acetone) to give the title compound as a white powder (0.41 g, 85 %): mp: 213–216 °C; ^1^H NMR (400 MHz, [D_6_]DMSO): *δ*=5.15 (2 H, s, C*H*_2_), 6.75 (2 H, AA′BB′, Ar*H*), 7.28 (1 H, dd, *J*=8.4, 2.4 Hz, Ar*H*), 7.44 (1 H, d, *J*=8.4 Hz, Ar*H*), 7.57–7.62 (2 H, m, Ar*H*), 7.68–7.79 (4 H, m, Ar*H*), 8.08–8.12 (2 H, m, Ar*H*), 8.81 ppm (2 H, s, 2×C*H*N); ^13^C NMR (100 MHz, [D_6_]DMSO): *δ*=56.9 (CH_2_), 103.1 (C), 113.7 (2×CH), 119.0 (C), 121.7 (CH), 123.3 (C), 126.6 (CH), 128.5 (C), 129.0 (2×CH), 129.9 (2×CH), 131.3 (C), 131.5 (CH), 134.0 (2×CH), 134.2 (CH), 143.2 (2×CH), 150.9 (C), 151.2 (C), 164.2 ppm (C); LC–MS (ES+): *t*_R_=1.40 min, *m*/*z* (%): 474.1 (100) [*M*+H]^+^; HRMS (ES+): *m*/*z* [*M*+H]^+^ calcd for C_23_H_17_N_5_O_2_Br: 474.0560, found: 474.0569.

**4-[(2-Bromo-4-hydroxybenzyl)(4*H*-1,2,4-triazol-4-yl)amino]benzonitrile (19 d)**: KOH (0.25 g, 4.46 mmol) was added to a suspension of **19 c** (0.35 g, 0.74 mmol) in MeOH (5 mL). After stirring for 2 h the reaction mixture was neutralised with 1 m aq. HCl and the product was extracted with EtOAc (3×30 mL). The combined organic layers were washed with sat. aq. NaHCO_3_ (60 mL) and brine (60 mL) then dried (MgSO_4_) and the solvent was removed in vacuo. The crude product was purified using the CombiFlash system (CH_2_Cl_2_/acetone) to give the title compound as a white solid (0.14 g, 52 %): mp: 233–235 °C; ^1^H NMR (400 MHz, [D_6_]DMSO): *δ*=4.97 (2 H, s, C*H*_2_), 6.63 (1 H, dd, *J*=8.4, 2.4 Hz, Ar*H*), 6.75 (2 H, AA′BB′, Ar*H*), 6.98 (1 H, d, *J*=2.4 Hz, Ar*H*), 7.03 (1 H, d, *J*=8.4 Hz, Ar*H*), 7.74 (2 H, AA′BB′, Ar*H*), 8.66 (2 H, s, 2×C*H*N), 9.97 ppm (1 H, s, O*H*); ^13^C NMR (100 MHz, [D_6_]DMSO): *δ*=56.4 (CH_2_), 102.8 (C), 113.6 (2×CH), 115.0 (CH), 119.0 (C), 119.4 (CH), 123.2 (C), 124.0 (C), 132.3 (CH), 133.9 (2×CH), 143.2 (2×CH), 151.2 (C), 158.4 ppm (C); LC–MS (ES+): *t*_R_=1.21 min, *m*/*z* (%): 370.0 (100) [*M*+H]^+^; HRMS (ES+): *m*/*z* [*M*+H]^+^ calcd for C_16_H_13_N_5_OBr: 370.0298, found: 370.0299.

**3-Bromo-4-{[(4-cyanophenyl)(4*H*-1,2,4-triazol-4-yl)amino]methyl}phenyl sulfamate (19)**: As general method B using ClSO_2_NH_2_ (0.6 m, 2.2 mL), DMA (2 mL) and **19 d** (0.105 g, 0.28 mmol). The crude product was purified using the CombiFlash system (CHCl_3_/acetone) to give the title compound as a white solid (0.094 g, 74 %): ^1^H NMR (400 MHz, [D_6_]DMSO): *δ*=5.13 (2 H, s, C*H*_2_), 6.71 (2 H, AA′BB′, Ar*H*), 7.22 (1 H, dd, *J*=8.4, 2.4 Hz, Ar*H*), 7.44 (1 H, d, *J*=2.4 Hz, Ar*H*), 7.55 (1 H, d, *J*=2.4 Hz, Ar*H*), 7.75 (2 H, AA′BB′, Ar*H*), 8.13 (2 H, s, N*H*_2_), 8.81 ppm (2 H, s, 2×C*H*N); ^13^C NMR (126 MHz, [D_6_]DMSO): *δ*=56.9 (CH_2_), 103.0 (C), 113.6 (2×CH), 119.0 (C), 121.7 (CH), 123.2 (C), 126.3 (CH), 131.4 (CH), 132.0 (C), 134.0 (2×CH), 143.3 (2×CH), 150.2 (C), 151.1 ppm (C); LC–MS (ES+): *t*_R_=1.19 min, *m*/*z* (%): 448.9 (100) [*M*+H]^+^; HRMS (ES+): *m*/*z* [*M*+H]^+^ calcd for C_16_H_14_N_6_O_3_SBr: 449.0032, found: 449.0026.

**(*E*)-*N*-[4-(Benzyloxy)benzylidene]aniline (20 a)**: A mixture of 4-benzyloxybenzaldehyde (5.31 g, 25.0 mmol), aniline (2.33 g, 25 mmol), *p*-TsOH⋅H_2_O (0.10 g) and EtOH (150 mL) was heated at reflux for 0.5 h. The reaction mixture was allowed to cool to room temperature and the crystals that formed were collected, washed with EtOH and dried under high vacuum to afford the title compound as light-yellow fine plates (6.11 g, 85 %): mp: 111–112 °C; ^1^H NMR (400 MHz, CDCl_3_) *δ*=5.14 (2 H, s, C*H*_2_), 7.07 (2 H, AA′BB′, Ar*H*), 7.17–7.25 (3 H, m, Ar*H*), 7.33–7.49 (7 H, m, Ar*H*), 7.87 (2 H, AA′BB′, Ar*H*), 8.39 ppm (1 H, s, C*H*); ^13^C NMR (100 MHz, CDCl_3_) *δ*=70.1 (CH2), 115.1 (2×CH), 120.9 (2×CH), 125.5 (CH), 127.5 (2×CH), 128.1 (CH), 128.6 (2×CH), 129.1 (2×CH), 129.5 (C), 130.5 (2×CH), 136.4 (C), 152.3 (C), 159.6 (CH), 161.4 ppm (C); Anal. calcd for C_20_H_17_NO: C 83.59, H 5.96, N 4.87, found: C 83.6, H 5.96, N 4.87.

***N*****-[4-(Benzyloxy)benzyl]aniline (20 b)**: NaBH_4_ (1.21 g, 31.99 mmol) was added to a cooled (0 °C) suspension of **20 a** (5.90 g, 20.53 mmol) in THF (50 mL) and EtOH (50 mL). The mixture was allowed to warm to room temperature and stirring was continued for 18 h. The reaction mixture was poured into H_2_O (250 mL) and the products were extracted with EtOAc (3×100 mL). The combined organic layers were washed with H_2_O (100 mL) and brine (50 mL), dried (MgSO_4_) and concentrated under reduced pressure. The residue was dissolved in CHCl_3_ and passed through a short SiO_2_ column (∼5 cm). The volatiles were removed in vacuo and the residue was recrystallised from CHCl_3_/PE to afford the title compound as fine colourless needles (5.57 g, 94 %): mp: 75–76 °C; ^1^H NMR (400 MHz, [D_6_]DMSO): *δ*=3.96 (1 H, s, N*H*), 4.28 (2 H, s, C*H*_2_), 5.09 (2 H, s, C*H*_2_), 6.67 (2 H, d, *J*=8.6 Hz, Ar*H*), 6.72–6.78 (1 H, m, Ar*H*), 6.90 (2 H, AA′BB′, Ar*H*), 7.18–7.23 (2 H, m, Ar*H*), 7.31 (2 H, AA′BB′, Ar*H*), 7.34–7.48 ppm (5 H, m, Ar*H*); ^13^C NMR (100 MHz, [D_6_]DMSO): *δ*=47.7 (CH_2_), 70.0 (CH_2_), 112.8 (2×CH), 114.9 (2×CH), 117.5 (CH), 127.4 (2×CH), 127.9 (CH), 128.6 (2×CH), 128.8 (2×CH), 129.2 (2×CH), 131.7 (C), 137.0 (C), 148.2 (C), 158.0 ppm (C); LRMS (ES+): *m*/*z* (%): 289.9 (25) [*M*+H]^+^, 196.8 (100); HRMS (ES+): *m*/*z* [*M*+H]^+^ calcd for C_20_H_20_NO: 290.1539, found: 290.1533; Anal. calcd for C_20_H_19_NO: C 83.01, H 6.62, N 4.84, found: C 83.0, H 6.56, N 4.80.

**1-[4-(Benzyloxy)benzyl]-1-phenylhydrazine (20 c)**: A solution of NaNO_2_ (2.07 g, 30 mmol) in H_2_O (10 mL) was slowly added to a cooled (0 °C) intensively stirred mixture of **20 b** (5.79 g, 20.0 mmol), CHCl_3_ (50 mL) and 2 m H_2_SO_4_ (40 mL). The mixture was stirred for 0.5 h and then the organic layer was separated, washed with brine (20 mL), dried (Na_2_SO_4_) and concentrated in vacuo. The residue was dissolved in THF (30 mL) and added to a suspension of LiAlH_4_ (1.44 g, 37.9 mmol) in THF (10 mL). The reaction mixture was stirred overnight at room temperature, then H_2_O (2 mL) and 5 m NaOH (3 mL) were added carefully. The inorganic salts were filtered off and washed with THF, the organic fraction was dried over Na_2_SO_4_ and the volatiles were removed in vacuo. The residue was purified using the CombiFlash *R*_f_ (CH_2_Cl_2_/MeOH) to afford the title compound as a light-yellow solid (5.23 g, 86 %): mp: 72–73 °C; ^1^H NMR (400 MHz, [D_6_]DMSO): *δ*=3.50 (2 H, s, *NH*_2_), 4.52 (2 H, s, C*H*_2_), 5.06 (2 H, s, C*H*_2_), 6.80–6.85 (1 H, m, Ar*H*), 6.96 (2 H, AA′BB′, Ar*H*), 7.11 (2 H, d, *J*=8.6 Hz, Ar*H*), 7.23 (2 H, AA′BB′, Ar*H*), 7.27–7.46 (7 H, m, Ar*H*); ^13^C NMR (100 MHz, [D_6_]DMSO): *δ*=59.8 (CH_2_), 70.0 (CH_2_), 113.8 (2×CH), 115.0 (2×CH), 118.6 (CH), 127.4 (2×CH), 128.0 (CH), 128.6 (2×CH), 129.0 (2×CH), 129.3 (2×CH), 129.6 (C), 137.0 (C), 151.8 (C), 158.2 ppm (C); LRMS (ES+): *m*/*z* (%): 305.0 (100) [*M*+H]^+^; HRMS (ES+): *m*/*z* [*M*+H]^+^ calcd for C_20_H_21_N_2_O: 305.1648, found: 305.1653; Anal. calcd for C_20_H_20_N_2_O: C 78.92, H 6.62, N 9.20, found: C 79.0, H 6.60, N 9.23.

***N*****-[4-(Benzyloxy)benzyl]-*N*-phenyl-4*H*-1,2,4-triazol-4-amine (20 d)**: Dimethylformamide diazine dihydrochloride^37^ (7.07 g, 32.86 mmol) was added to a solution of **20 c** (5.00 g, 16.43 mmol) in pyridine (10 mL) and the mixture was heated at reflux for 2 h. After cooling to room temperature EtOAc (60 mL) and H_2_O (30 mL) were added, the organic layer was separated, washed with 2 m KHSO_4_ solution (2×30 mL) and brine (30 mL), dried (Na_2_SO_4_) and concentrated under reduced pressure. The residue was recrystallised from MeOH to afford the title compound as pale-yellow plates (3.12 g, 53 %): mp: 165–167 °C; ^1^H NMR (400 MHz, [D_6_]DMSO): *δ*=4.87 (2 H, s, C*H*_2_), 5.05 (2 H, s, C*H*_2_), 6.77 (2 H, AA′BB′, Ar*H*), 6.92–6.96 (2 H, m, Ar*H*), 6.99–7.04 (1 H, m, Ar*H*), 7.24 (2 H, AA′BB′, Ar*H*), 7.29–7.46 (7 H, m, Ar*H*), 8.74 ppm (2 H, s, 2×C*H*N); ^13^C NMR (100 MHz, [D_6_]DMSO): *δ*=57.3 (CH_2_), 69.2 (CH_2_), 114.7 (2×CH), 114.8 (2×CH), 122.1 (CH), 127.4 (C), 127.8 (2×CH), 127.9 (CH), 128.4 (2×CH), 129.4 (2×CH), 130.1 (2×CH), 136.9 (C), 143.4 (2×CH), 148.8 (C), 158.0 ppm (C); LRMS (ES+): *m*/*z* (%): 357.1 (100) [*M*+H]^+^; HRMS (ES+): *m*/*z* [*M*+H]^+^ calcd for C_22_H_21_N_4_O: 357.1710, found: 357.1717; Anal. calcd for C_22_H_20_N_4_O: C 74.14, H 5.66, N 15.72, found: C 74.2, H 5.61, N 15.7.

**4-{[Phenyl(4*H*-1,2,4-triazol-4-yl)amino]methyl}phenol (20 e)**: As general method A using 5 % Pd/C (0.10 g), **20 d** (1.36 g, 3.80 mmol) and THF/MeOH (1:1, 40 mL). The crude product was purified using the CombiFlash system (CHCl_3_/acetone) to give the title compound as a white solid (0.74 g, 74 %): mp: 191–193 °C; ^1^H NMR (400 MHz, [D_6_]DMSO): *δ*=4.78 (2 H, s, C*H*_2_), 6.65 (2 H, AA′BB′, Ar*H*), 6.72–6.76 (2 H, m, Ar*H*), 6.97–7.02 (1 H, m, Ar*H*), 7.07 (2 H, AA′BB′, Ar*H*), 7.28–7.34 (2 H, m, Ar*H*), 8.67 (2 H, s, 2×C*H*N), 9.43 ppm (1 H, s, O*H*); ^13^C NMR (100 MHz, [D_6_]DMSO): *δ*=57.3 (CH_2_), 114.7 (2×CH), 115.2 (2×CH), 122.0 (CH), 125.2 (C), 129.4 (2×CH), 130.1 (2×CH), 143.4 (2×CH), 148.9 (C), 157.0 ppm (C); LRMS (ES+): *m*/*z* (%): 266.8 (100) [*M*+H]^+^; HRMS (ES+): *m*/*z* [*M*+H]^+^ calcd for C_15_H_15_N_4_O: 267.1240, found: 267.1240; Anal. calcd for C_15_H_14_N_4_O: C 67.65, H 5.30, N 21.04, found: C 67.5, H 5.16, N, 21.2.

**4-{[Phenyl(4*H*-1,2,4-triazol-4-yl)amino]methyl}phenyl sulfamate (20)**: As general method B using ClSO_2_NH_2_ (0.63 m, 5.0 mL), DMA (5 mL) and **20 e** (0.266 g, 1.00 mmol). The title compound (0.093 g, 27 %) was obtained as a light-yellow solid which was recrystallised from a small amount of acetone/hexane to give colourless crystals containing 1 equiv acetone: mp: 140–142 °C; ^1^H NMR (400 MHz, [D_6_]DMSO): *δ*=4.97 (2 H, s, C*H*_2_), 6.65–6.75 (2 H, m, Ar*H*), 6.95–7.05 (1 H, m, Ar*H*), 7.21 (2 H, AA′BB′, Ar*H*), 7.28–7.38 (2 H, m, Ar*H*), 7.42 (2 H, AA′BB′, Ar*H*), 8.03 (2 H, s, N*H*_2_), 8.82 ppm (2 H, s, 2×C*H*N); ^13^C NMR (100 MHz, [D_6_]DMSO): *δ*=57.3 (CH_2_), 114.7 (2×CH), 122.1 (CH), 122.2 (2×CH), 129.5 (2×CH), 129.7 (2×CH), 134.0 (C), 143.4 (2×CH), 148.6 (C), 149.7 ppm (C); LRMS (ES+): *m*/*z* (%): 346.0 (100) [*M*+H]^+^; HRMS (ES+): *m*/*z* [*M*+H]^+^ calcd for C_15_H_16_N_5_O_3_S: 346.0968, found: 346.0961; Anal. calcd for C_15_H_15_N_5_O_3_S⋅C_3_H_6_O: C 53.59, H 5.25, N 17.36, found: C 53.3, H 5.36, N 17.6.

**(4-Benzyloxybenzylidene)-(3,5-difluorophenyl)amine (21 a)**: A mixture of 3,5-difluoroaniline (1.94 g, 15 mmol), 4-benzyloxybenzaldehyde (3.18 g, 15 mmol) and *p*-TsOH⋅H_2_O (0.10 g) in EtOH (50 mL) was heated at reflux for 1 h. The reaction mixture was allowed to cool to room temperature and the crystals that formed were collected, washed with EtOH and dried under high vacuum to afford the title compound as colourless plates (3.88 g, 80 %): mp: 78–79 °C; ^1^H NMR (400 MHz, [D_6_]DMSO): *δ*=5.14 (2 H, s, C*H*_2_), 6.60–6.73 (3 H, m, Ar*H*), 7.05 (2 H, AA′BB′, Ar*H*), 7.35–7.48 (5 H, m, Ar*H*), 7.82 (2 H, AA′BB′, Ar*H*), 8.31 ppm (1 H, s, C*H*); ^13^C NMR (100 MHz, [D_6_]DMSO): *δ*=70.1 (CH_2_), 100.6 (t, CH), 104.1 (2×CH, m), 115.1 (2×CH), 127.5 (2×CH), 128.2 (CH), 128.7 (2×CH), 130.8. (C), 130.9 (2×CH), 136.2 (C), 155.0 (C, t), 161.1 (CH), 161.9 (C), 163.2 ppm (2×C, dd); LRMS (ES+): *m*/*z* (%): 324.5 (100) [*M*+H]^+^; Anal. calcd for C_20_H_15_F_2_NO: C 74.29, H 4.68, N 4.33, found: C 74.3, H 4.69, N 4.14.

**(*E*)-(4-Benzyloxybenzyl)-(3,5-difluorophenyl)amine (21 b)**: NaBH_4_ (1.0 g, 26.3 mmol) was added to a cooled (0 °C) suspension of **21 a** (3.52 g, 10.89 mmol) in THF (20 mL) and EtOH (20 mL). The mixture was allowed to warm to room temperature and stirring was continued for 18 h. The mixture was poured into H_2_O (100 mL) and extracted with EtOAc (3×50 mL). The combined organic fractions were washed with H_2_O (50 mL) and brine (50 mL), dried (Na_2_SO_4_) and concentrated under reduced pressure. The residue was purified by flash column chromatography (EtOAc) to afford the title compound as a white solid (3.37 g, 95 %): mp: 79–80 °C; ^1^H NMR (400 MHz, [D_6_]DMSO): *δ*=4.20 (2H s, C*H*_2_), 5.07 (2 H, s, CH_2_), 6.20–6.28 (3 H, m, Ar*H*), 6.96 (2 H, AA′BB′, Ar*H*), 7.25 (2 H, AA′BB′, Ar*H*), 7.32–7.45 ppm (5 H, m, Ar*H*); ^13^C NMR (100 MHz, [D_6_]DMSO): *δ*=47.5 (CH_2_), 70.1 (CH_2_), 92.5 (t, CH), 95.4 (2×CH, m), 115.1 (2×CH), 127.4 (2×CH), 128.0 (CH), 128.6 (2×CH), 128.8 (2×CH), 130.4 (C) 136.9 (C), 150.2 (C, t), 158.3 (C), 163.5 ppm (2×C, dd); HRMS (EI): *m*/*z* [*M*]^+^ calcd for C_20_H_17_F_2_NO: 325.1273, found: 325.1276; Anal. calcd for C_20_H_17_F_2_NO: C 73.83, H 5.27, N 4.31, found: C 73.8, H 5.26, N 4.15.

***N*****-(4-Benzyloxybenzyl)-*N*-(3,5-difluorophenyl)hydrazine (21 c)**: A solution of NaNO_2_ (1.04 g, 15 mmol) in H_2_O (10 mL) was slowly added to a cooled (0 °C) intensively stirred mixture of **21 b** (3.25 g, 10.0 mmol), CHCl_3_ (50 mL) and 2 m H_2_SO_4_ (20 mL). The mixture was stirred for 1 h and then the organic layer was separated, washed with brine (20 mL), dried (Na_2_SO_4_) and concentrated in vacuo. The residue was dissolved in THF (20 mL) and added to a suspension of LiAlH_4_ (0.25 g) in THF (30 mL). The reaction mixture was stirred overnight at room temperature, then H_2_O (2 mL) and 5 m NaOH (3 mL) were added carefully. The inorganic salts were filtered off and washed with THF, the organic fraction was dried over Na_2_SO_4_ and the volatiles were removed in vacuo. The residue was purified by flash column chromatography (CHCl_3_) to afford the title compound as a white solid (2.45 g, 72 %): mp: 94–96 °C; ^1^H NMR (400 MHz, CDCl_3_): *δ*=3.57 (2 H, br s, N*H*_2_), 4.56 (2 H, s, C*H*_2_), 5.15 (2 H, s, C*H*_2_), 6.28–6.34 (1 H, m, Ar*H*), 6.69–6.74 (2 H, m, Ar*H*), 7.07 (2 H, AA′BB′, Ar*H*), 7.27 (2 H, AA′BB′, Ar*H*), 7.42–7.56 ppm (5 H, m, Ar*H*); ^13^C NMR (100 MHz, [D_6_]DMSO): *δ*=58.2 (CH_2_), 69.9 (CH_2_), 92.6 (t, CH), 95.8 (2×CH, m), 115.1 (2×CH), 127.4 (2×CH), 127.9 (CH), 128.3 (C), 128.5 (2×CH), 128.9 (2×CH), 136.7 (C), 153.7 (C, t), 158.3 (C), 163.8 ppm (2×C, dd); LRMS (ES^−^): *m*/*z* (%): 339.4 (100) [*M*−H]^−^; HRMS (ES+): *m*/*z* [*M*+H]^+^ calcd for C_20_H_19_F_2_N_2_O: 341.1460, found 341.1452; Anal. calcd for C_20_H_18_F_2_N_2_O: C 70.58, H 5.33, N 8.23, found: C 70.7, H 5.32, N 8.29.

**(4-Benzyloxybenzyl)-(3,5-difluorophenyl)-[1,2,4]triazol-4-ylamine (21 d)**: Dimethylformamide diazine dihydrochloride (2.15 g, 10.0 mmol) was added to a solution of **21 c** (1.70 g, 5.0 mmol) in pyridine (5 mL) and the mixture was heated at reflux for 2 h. After cooling to room temperature EtOAc (60 mL) and H_2_O (30 mL) were added, the organic layer was separated, washed with 2 m KHSO_4_ solution (2×30 mL) and brine (30 mL), dried (Na_2_SO_4_) and concentrated under reduced pressure. The residue was purified by flash column chromatography (CHCl_3_/acetone) to afford the title compound (1.37 g, 70 %) as a colourless solid, which recrystallised from CHCl_3_/hexane to give pale-yellow plates: mp: 142–143 °C; ^1^H NMR (400 MHz, CDCl_3_): *δ*=4.68 (2 H, s, C*H*_2_), 5.01 (2 H, s, C*H*_2_), 6.16–6.20 (2 H, m, Ar*H*), 6.43–6.49 (1 H, m, Ar*H*), 6.90 (2 H, AA′BB′, Ar*H*), 7.07 (2 H, AA′BB′, Ar*H*), 7.31–7.42 (5 H, m, Ar*H*), 8.03 ppm (2 H, s, 2×C*H*N); ^13^C NMR (100 MHz, [D_6_]DMSO): *δ*=57.9 (CH_2_), 70.0 (CH_2_), 97.5 (m, CH, 2×CH), 115.4 (2×CH), 125.5 (C), 127.5 (2×CH), 128.1 (CH), 128.6 (2×CH), 129.8 (2×CH), 136.4 (C), 142.6 (2×CH), 149.8 (C, t), 159.2 (C), 163.8 (2×C, dd); ^19^F NMR (376.4 MHz, [D_6_]DMSO): *δ*=−106.8 ppm (m); LRMS (ES^−^): *m*/*z* (%): 391.3 (10) [*M*−H]^−^; HRMS (ES+): *m*/*z* [*M*+H]^+^ calcd for C_22_H_19_F_2_N_4_O: 393.1521, found: 393.1526; Anal. calcd for C_22_H_18_F_2_N_4_O: C 67.34, H 4.62, N 14.28, found: C 67.2, H 4.74, N 14.2.

**4-{[(3,5-Difluorophenyl)(4*H*-1,2,4-triazol-4-yl)amino]methyl}phenol (21 e)**: As general method A using 5 % Pd/C (0.10 g), **21 d** (1.02, 2.60 mmol) and THF/MeOH (1:1, 40 mL). The crude product was dissolved in acetone and Et_2_O was added. The precipitate was filtered off and dried under high vacuum to afford the title compound as a white solid (0.668 g, 85 %): mp: 214–217 °C; ^1^H NMR (400 MHz, [D_6_]DMSO): *δ*=4.83 (2 H, s, C*H*_2_), 6.36 (2 H, s, Ar*H*), 6.65 (2 H, AA′BB′, Ar*H*), 6.80–6.87 (1 H, m, Ar*H*), 7.03 (2 H, AA′BB′, Ar*H*), 8.67 (2 H, s, 2×C*H*N), 9.49 ppm (1 H, s, O*H*); ^13^C NMR (100 MHz, [D_6_]DMSO): *δ*=57.0 (CH_2_), 96.5 (t, CH), 97.7 (m, 2×CH), 115.3 (2×CH), 124.4 (C), 130.3 (2×CH), 143.4 (2×CH), 151.2 (C, t), 159.2 (C), 163.3 ppm (2×C, dd); ^19^F NMR (376.4 MHz, [D_6_]DMSO): *δ*=−108.4 ppm (m); LRMS (ES+): *m*/*z* (%): 303.4 (100) [*M*+H]^+^; HRMS (ES+): *m*/*z* [*M*+H]^+^ calcd for C_15_H_13_F_2_N_4_O: 303.1052, found: 303.1058; Anal. calcd for C_15_H_12_F_2_N_4_O: C 59.90, H 4.00, N 18.53, found: C 59.4, H 4.14, N 18.7.

**4-{[(3,5-Difluorophenyl)(4*H*-1,2,4-triazol-4-yl)amino]methyl}phenyl sulfamate (21)**: As general method B using ClSO_2_NH_2_ (0.65 m, 5.0 mL), DMA (5 mL) and **21 e** (0.302 g, 1.00 mmol). The crude product was recrystallised from a small amount of acetone to afford the title compound as colourless crystals (0.143 g, 37 %): mp: 178–180 °C; ^1^H NMR (400 MHz, [D_6_]DMSO): *δ*=5.03 (2 H, s, C*H*_2_), 6.35–6.45 (2 H, m, Ar*H*), 6.82–6.90 (1 H, m, Ar*H*), 7.23 (2 H, AA′BB′, Ar*H*), 7.39 (2 H, AA′BB′, Ar*H*), 8.05 (2 H, s, N*H*_2_), 8.81 (2 H, s, 2×C*H*N); ^13^C NMR (100 MHz, [D_6_]DMSO): *δ*=57.0 (CH_2_), 96.8 (t, CH), 97.6 (m, 2×CH), 122.3 (2×CH), 130.0 (2×CH), 133.2 (C), 143.3 (2×CH), 149.9 (C), 151.0 (C, t), 163.3 ppm (2×C, dd); ^19^F NMR (376.4 MHz, [D_6_]DMSO): *δ*=−108.2 ppm (m); LRMS (ES^−^): *m*/*z* (%): 380.2 (100) [*M*−H]^−^; HRMS (ES+): *m*/*z* [*M*+H]^+^ calcd for C_15_H_14_F_2_N_5_O_3_S: 382.0780, found: 382.0789; Anal. calcd for C_15_H_13_F_2_N_5_O_3_S: C 47.24, H 3.44, N 18.36, found: C 47.2, H 3.42, N 18.6.

**(*E*)-(4-Benzyloxybenzylidene)-(2,2-difluorobenzo[1,3]dioxol-5-yl)amine (22 a)**: A mixture of 5-amino-2,2-difluoro-1,3-benzodioxole (2.60 g, 15 mmol), 4-benzyloxybenzaldehyde (3.18 g, 15 mmol) and *p*-TsOH⋅H_2_O (0.10 g) in EtOH (50 mL) was heated at reflux for 1 h. The reaction mixture was allowed to cool to room temperature and the crystals that formed were collected, washed with EtOH and dried under high vacuum to afford the title compound as colourless plates (4.30 g, 78 %): mp: 114–116 °C; ^1^H NMR (400 MHz, CDCl_3_): *δ*=5.20 (2 H, s, C*H*_2_), 6.90 (1 H, dd, *J*=8.6, 2.4 Hz, Ar*H*), 6.96 (1 H, d, *J*=2.4 Hz, Ar*H*), 7.02–7–08 (3 H, m, Ar*H*), 7.33–7.46 (5 H, m, Ar*H*), 7.83 (2 H, AA′BB′, Ar*H*), 8.33 ppm (1 H, C*H*); ^13^C NMR (100 MHz, CDCl_3_): *δ*=70.1 (CH_2_), 102.7 (C), 109.5 (CH), 115.1 (2×CH), 116.2 (CH), 127.5 (2×CH), 128.2 (CH), 128.7 (2×CH), 129.0 (C), 130.6 (2×CH), 131.8 (C, t, ^1^*J*_C-F_=255 Hz), 136.3 (C), 141.6 (C), 144.2 (C), 148.6 (C), 159.8 (CH), 161.6 ppm (C); ^19^F NMR (376.4 MHz, CDCl_3_): *δ*=−49.8 ppm (s); LRMS (ES+): *m*/*z* (%): 368.4 (100) [*M*+H]^+^; HRMS (ES+): *m*/*z* [*M*+H]^+^ calcd for C_21_H_15_F_2_NO_3_: 368.1093, found: 368.1099; Anal. calcd for C_21_H_15_F_2_NO_3_: C 68.66, H 4.12, N 3.81, found: C 68.6, H 4.11, N 3.70.

**(4-Benzyloxybenzyl)-(2,2-difluorobenzo[1,3]dioxol-5-yl)amine (22 b)**: NaBH_4_ (1.0 g, 26.3 mmol) was added to a cooled (0 °C) suspension of **22 a** (4.02 g, 10.94 mmol) in THF (20 mL) and EtOH (20 mL). The mixture was allowed to warm to room temperature and stirring was continued for 18 h. The mixture was poured into H_2_O (100 mL) and extracted with EtOAc (3×50 mL). The combined organic fractions were washed with H_2_O (50 mL) and brine (50 mL), dried (Na_2_SO_4_) and concentrated under reduced pressure. The residue was purified by flash column chromatography (EtOAc) to afford the title compound as a white solid (3.89 g, 96 %): mp: 63–64 °C; ^1^H NMR (400 MHz, CDCl_3_): *δ*=3.97 (1 H, s, N*H*), 4.19 (2 H, s, C*H*_2_), 5.06 (2 H, s, C*H*_2_), 6.24 (1 H, dd, *J*=8.6, 2.3 Hz, Ar*H*), 6.36 (1 H, d, *J*=2.3 Hz, Ar*H*), 6.83 (1 H, d, *J*=8.6 Hz, Ar*H*), 6.96 (2 H, AA′BB′, Ar*H*), 7.26 (2 H, AA′BB′, Ar*H*), 7.30–7.45 ppm (5 H, m, Ar*H*); ^13^C NMR (100 MHz, CDCl_3_): *δ*=48.3 (CH_2_), 70.0 (CH_2_), 95.3 (CH), 106.5 (CH), 109.7 (CH), 115.0 (2×CH), 127.4 (2×CH), 128.0 (CH), 128.6 (2×CH), 128.7 (2×CH), 130.9 (C), 131.7 (C, t, ^1^*J*_C-F_=253 Hz), 135.8 (C), 136.9 (C), 144.6 (C), 145.2 (C), 158.2 ppm (C); LRMS (APCI^−^): *m*/*z* (%): 368.4 (100) [*M*−H]^−^; HRMS (ES+): *m*/*z* [*M*+Na]^+^ calcd for C_21_H_17_F_2_NO_3_Na: 392.1069, found: 392.1065; Anal. calcd for C_21_H_17_F_2_NO_3_: C 68.29, H 4.64, N 3.79, found: C 68.3, H 4.63, N, 3.62.

***N*****-(4-Benzyloxybenzyl)-*N*-(2,2-difluorobenzo[1,3]dioxol-5-yl)hydrazine (22 c)**: A solution of NaNO_2_ (1.04 g, 15 mmol) in H_2_O (10 mL) was slowly added to a cooled (0 °C) intensively stirred mixture of **22 b** (3.69 g, 10.0 mmol), CHCl_3_ (50 mL) and 2 m H_2_SO_4_ (20 mL). The mixture was stirred for 1 h and then the organic layer was separated, washed with brine (20 mL), dried (Na_2_SO_4_) and concentrated in vacuo. The residue was dissolved in THF (20 mL) and added to a suspension of LiAlH_4_ (0.25 g) in THF (30 mL). The reaction mixture was stirred overnight at room temperature, then H_2_O (2 mL) and 5 m NaOH (3 mL) were added carefully. The inorganic salts were filtered off and washed with THF, the organic fraction was dried over Na_2_SO_4_ and the volatiles were removed in vacuo. The residue was purified by flash column chromatography (CHCl_3_) to afford the title compound as a white solid (2.88 g, 75 %): mp: 80–81 °C; ^1^H NMR (400 MHz, [D_6_]DMSO): *δ*=3.50 (2 H, br s, N*H*_2_), 4.43 (2 H, s, C*H*_2_), 5.06 (2 H, s, C*H*_2_), 6.71 (1 H, dd, *J*=8.6, 2.3 Hz, Ar*H*), 6.89 (1 H, d, *J*=8.6 Hz, Ar*H*), 6.95 (2 H, AA′BB′, Ar*H*), 6.99 (1 H, d, *J*=2.3 Hz, Ar*H*), 7.18 (2 H, AA′BB′, Ar*H*), 7.30–7.45 ppm (5 H, m, Ar*H*); ^13^C NMR (100 MHz, [D_6_]DMSO): *δ*=60.7 (CH_2_), 70.0 (CH_2_), 97.7 (CH), 108.1 (CH), 109.1 (CH), 115.1 (2×CH), 127.5 (2×CH), 128.0 (CH), 128.5 (C), 128.6 (2×CH), 129.4 (2×CH), 131.8 (C, t, ^1^*J*_CF_=253 Hz), 136.7 (C), 136.8 (C), 144.5 (C), 149.0 (C), 158.4 ppm (C); HRMS (ES+): *m*/*z* [*M*+H]^+^ calcd for C_21_H_19_F_2_N_2_O_3_: 385.1358, found: 385.1361; Anal. calcd for C_21_H_18_F_2_N_2_O_3_: C 65.62, H 4.72, N 7.29, found: C 65.4, H 4.72, N 7.37.

**(4-Benzyloxybenzyl)-(2,2-difluorobenzo[1,3]dioxol-5-yl)-[1,2,4]triazol-4-ylamine (22 d)**: Dimethylformamide diazine dihydrochloride (2.47 g, 11.5 mmol) was added to a solution of **22 c** (2.21 g, 5.75 mmol) in pyridine (5 mL) and the mixture was heated at reflux for 2 h. After cooling to room temperature EtOAc (60 mL) and H_2_O (30 mL) were added, the organic layer was separated, washed with 2 m KHSO_4_ solution (2×30 mL) and brine (30 mL), dried (Na_2_SO_4_) and concentrated under reduced pressure. The residue was purified by flash column chromatography (CHCl_3_/acetone) to afford the title compound as a colourless solid, which recrystallised from CHCl_3_/hexane to give colourless plates (1.41 g, 56 %): mp: 112–114 °C; ^1^H NMR (400 MHz, CDCl_3_): *δ*=4.63 (2 H, s, C*H*_2_), 4.99 (2 H, s, C*H*_2_), 6.47 (1 H, dd, *J*=9.0, 2.7 Hz, Ar*H*), 6.57 (1 H, d, *J*=2.7 Hz, Ar*H*), 6.89 (2 H, AA′BB′, Ar*H*), 6.96 (1 H, d, *J*=9.0 Hz, Ar*H*), 7.08 (2 H, AA′BB′, Ar*H*), 7.31–7.41 (5 H, m, Ar*H*), 8.06 ppm (2 H, s, 2×C*H*N); ^13^C NMR (100 MHz, CDCl_3_): *δ*=59.0 (CH_2_), 69.9 (CH_2_), 99.0 (CH), 109.8 (CH), 110.3 (CH), 115.2 (2×CH), 125.8 (C), 127.4 (2×CH), 128.0 (CH), 128.5 (2×CH), 129.9 (2×CH), 131.6 (C, t), 136.4 (C), 139.5 (C), 142.4 (2×CH), 144.5 (C), 144.6 (C), 159.0 ppm (C); LRMS (ES^−^): *m*/*z* (%): 435.5 (100) [*M*−H]^−^; HRMS (ES+): *m*/*z* [*M*+H]^+^ calcd for C_23_H_19_F_2_N_4_O_3_: 437.1420, found: 437.1420; Anal. calcd for C_23_H_18_F_2_N_4_O_3_: C 63.30, H 4.16, N 12.84, found: C 63.0, H 4.19, N 12.9.

**4-{[(2,2-Difluorobenzo[1,3]dioxol-5-yl)-[1,2,4]triazol-4-ylamino]methyl}phenol (22 e)**: As general method A using 5 % Pd/C (0.10 g), **22 d** (1.20, 2.76 mmol) and THF/MeOH (1:1, 40 mL). The crude product was recrystallised from acetone/Et_2_O to afford the title compound (0.88 g, 92 %) as a white solid; mp: 182–185 °C; ^1^H NMR (400 MHz, [D_6_]DMSO): *δ*=4.75 (2 H, s, C*H*_2_), 6.48 (1 H, dd, *J*=8.6, 2.4 Hz, Ar*H*), 6.65 (2 H, AA′BB′, Ar*H*), 7.04 (2 H, AA′BB′, Ar*H*), 7.16 (1 H, d, *J*=2.4 Hz, Ar*H*), 7.35 (1 H, d, *J*=8.6 Hz, Ar*H*), 8.67 (2 H, s, 2×C*H*N), 9.47 ppm (1 H, s, O*H*); ^13^C NMR (100 MHz, [D_6_]DMSO): *δ*=58.0 (CH_2_), 99.5 (CH), 110.5 (CH), 111.2 (CH), 115.3 (2×CH), 124.8 (C), 130.3 (2×CH), 131.4 (C, t), 138.1 (C), 143.2 (2×CH), 143.5 (C), 146.0 (C), 157.2 ppm (C); ^19^F NMR (376.4 MHz, [D_6_]DMSO): *δ*=−49.1 ppm (s); LRMS (ES+): *m*/*z* (%): 369.3 (55) [*M*+Na]^+^, 347.3 (20) [*M*+H]^+^, 278.2 (100); HRMS (ES+): *m*/*z* [*M*+H]^+^ calcd for C_16_H_13_F_2_N_4_O_3_: 347.0950, found: 347.0959; Anal. calcd for C_16_H_12_F_2_N_4_O_3_: C 55.49, H 3.49, N, 16.18, found: C 55.5, H 3.49, N 16.4.

**4-{[(2,2-Difluorobenzo[*d*][1,3]dioxol-5-yl)(4*H*-1,2,4-triazol-4-yl)amino]methyl}phenyl sulfamate (22)**: As general method B using ClSO_2_NH_2_ (0.65 m, 5.0 mL), DMA (5 mL) and **22 e** (0.35 g, 1.00 mmol). The crude product was purified by flash column chromatography (CHCl_3_/acetone) to afford the title compound as a white solid (0.32 g, 75 %): mp: 132–134 °C; ^1^H NMR (400 MHz, [D_6_]DMSO): *δ*=4.94 (2 H, s, C*H*_2_), 6.50 (1 H, dd, *J*=9.0, 2.5 Hz, Ar*H*), 7.17 (1 H, d, *J*=2.5 Hz, Ar*H*), 7.21 (2 H, AA′BB′, Ar*H*), 7.35 (1 H, d, *J*=9.0 Hz, Ar*H*), 7.40 (2 H, AA′BB′, Ar*H*), 8.04 (2 H, s, N*H*_2_), 8.81 ppm (2 H, s, 2×C*H*N); ^13^C NMR (100 MHz, [D_6_]DMSO): *δ*=57.9 CH_2_), 99.6 (CH), 110.6 (CH), 111.3 (CH), 122.2 (2×CH), 130.0 (2×CH), 131.4 (C, t), 133.4 (C), 138.3 (C), 143.1 (2×CH), 143.6 (C), 145.8 (C), 149.8 ppm (C); ^19^F NMR (376.4 MHz, [D_6_]DMSO): *δ*=−49.1 ppm (s); LRMS (ES^−^): *m*/*z* (%): 424.4 (100) [*M*−H]^−^; HRMS (ES+): *m*/*z* [*M*+H]^+^ calcd for C_16_H_14_F_2_N_5_O_5_S: 426.0678, found: 426.0686; Anal. calcd for C_16_H_13_F_2_N_5_O_5_S: C 45.18, H 3.08, N 16.46, found: C 45.0, H 3.10, N 16.5.

**4-(2-Thioxo-2,3-dihydro-1*H*-imidazol-1-ylamino)benzonitrile (23)**: A mixture of 4-cyanophenylhydrazine hydrochloride (5.00 g, 29.48 mmol), 2-isothiocyanato-1,1-dimethoxyethane (5.0 g, 33.97 mmol) in AcOH (50 mL) and H_2_O (5 mL) was heated at 100 °C for 1 hour. The mixture was poured into H_2_O (300 mL) after cooling to room temperature. The precipitate was collected by filtration, washed with H_2_O and dried under high vacuum to afford **23** as a yellow powder (5.12 g, 80 %): mp:>250 °C; ^1^H NMR (400 MHz, [D_6_]DMSO): *δ*=6.55 (AA′BB′, 2 H), 7.02 (t, *J*=2.3 Hz, 1 H), 7.24 (t, *J*=2.3 Hz, 1 H), 7.63 (AA′BB′, 2 H), 9.87 (s, 1 H), 12.45 ppm (s, 1 H); ^13^C NMR (100.6 MHz, [D_6_]DMSO): *δ*=100.8, 112.6, 113.8, 119.7, 120.3, 133.6, 151.0, 162.3 ppm; LRMS (ES+): *m*/*z* (%): 216.6 (100) [*M*+H]^+^); HRMS (ES+): *m*/*z* [*M*+H]^+^ calcd for C_10_H_9_N_4_S: 217.0542, found: 217.0541; Anal. calcd for C_10_H_8_N_4_S: C 55.54, H 3.73, N 25.91, found: C 55.3, H 3.58, N 25.9.

**4-(1*H*-Imidazol-1-ylamino)benzonitrile (24)**: To a suspension of **23** (2.99 g, 13.83 mmol) in AcOH (10 mL) was added 50 % H_2_O_2_ (2.0 mL) slowly via syringe (exothermic reaction) with ice cooling. The resulting clear red-brown solution was stirred for 0.5 h before it was diluted with H_2_O (10 mL), neutralised with 5 m NaOH and treated with 1 m NaSO_3_ (5 mL). The mixture was extracted with EtOAc (3×30 mL), the combined extracts were dried (Na_2_SO_4_) and concentrated under reduced pressure. The residue was purified by chromatography (ISCO CombiFlash; gradient: CH_2_Cl_2_ to CH_2_Cl_2_/MeOH (90:10), 40 g SiO_2_ column) to give crude **24** as an orange solid. The solid was dissolved in EtOAc and treated with activated charcoal. The charcoal was removed by filtration and the volatiles were removed under reduced pressure. The residue was recrystallised from EtOAc/hexane to afford **24** as a light-yellow solid (1.501 g, 59 %): mp: 157–159 °C; ^1^H NMR (400 MHz, [D_6_]DMSO): *δ*=6.49 (AA′BB′, 2 H), 7.06 (t, *J*=1.2 Hz, 1 H), 7.33 (t, *J*=1.2 Hz, 1 H), 7.65 (AA′BB′, 2 H), 7.82 (s, 1 H), 10.11 ppm (s, 1 H); ^13^C NMR (100.6 MHz, [D_6_]DMSO): *δ*=104.4, 112.0, 119.5, 120.7, 128.0, 134.0, 137.6, 151.9 ppm; LRMS (ES+): *m*/*z* (%): 184.6 (100) [*M*+H]^+^; HRMS (ES+): *m*/*z* [*M*+H]^+^ calcd for C_10_H_9_N_4_O: 185.0822, found: 185.0819.

**[4-(Benzyloxy)-2-fluorophenyl]methanol (25 a)**: A mixture of **11 b** (0.85 g, 5.98 mmol), benzyl bromide (1.13 g, 6.58 mmol), K_2_CO_3_ (5.00 g, 36.23 mmol) and DMF (20 mL) was stirred intensively for 48 h. The mixture was poured into H_2_O (100 mL) and the product was extracted with EtOAc (3×50 mL). The organic layers were dried (MgSO_4_) and concentrated in vacuo. The residue was purified using the CombiFlash *R*_f_ (EtOAc/PE) to afford the title compound as a colourless oil which solidified upon standing (1.17 g, 84 %): mp: 67–68 °C; ^1^H NMR (400 MHz, CDCl_3_): *δ*=1.78 (1 H, t, *J*=6.1 Hz, O*H*), 4.67 (2 H, d, *J*=6.1 Hz, C*H*_2_), 5.05 (2 H, s, C*H*_2_), 6.68–6.79 (2 H, m, Ar*H*), 7.25–7.45 ppm (6 H, m, Ar*H*); LRMS (ES+): *m*/*z* (%): 214.8 (100) [*M*−OH]^+^.

**4-(Benzyloxy)-1-(chloromethyl)-2-fluorobenzene (25 b)**: SOCl_2_ (1.0 mL) was added to a solution of **25 a** (1.12 g, 4.82 mmol) in CH_2_Cl_2_ (20 mL) and the resulting solution was stirred for 2 h. Then H_2_O (10 mL) was added, the organic layer was separated, washed with brine (10 mL), dried (MgSO_4_) and concentrated in vacuo. The residue was purified using the CombiFlash *R*_f_ (EtOAc/PE) to afford the title compound as a colourless oil (1.02 g, 85 %): ^1^H NMR (400 MHz, [D_6_]DMSO): *δ*=4.60 (2 H, s, C*H*_2_), 5.05 (2 H, s, C*H*_2_), 6.67–6.77 (2 H, m, Ar*H*), 7.30–7.43 ppm (6 H, m, Ar*H*); LRMS (ES+): *m*/*z* (%): 250.7 (100) [*M*+H]^+^.

**4-{[4-(Benzyloxy)-2-fluorobenzyl](1*H*-imidazol-1-yl)amino}benzonitrile (25 c)**: NaH (60 % dispersion in mineral oil, 0.12 g, 3.0 mmol) was added to a solution of **24** (0.55 g, 3.0 mmol) in DMF (10 mL). After stirring the mixture for 0.5 h, **25 b** (0.75 g, 3.0 mmol) was added and stirring was continued for 18 h. The reaction mixture was diluted with EtOAc (60 mL), washed with H_2_O (3×30 mL) and brine (30 mL), dried (Na_2_SO_4_) and concentrated in vacuo. The residue was purified using the CombiFlash *R*_f_ (EtOAc/PE) to afford an orange solid, which was recrystallised from EtOAc/PE to give the title compound as colourless crystals (1.01 g, 85 %): mp: 132–134 °C; ^1^H NMR (400 MHz, [D_6_]DMSO): *δ*=4.99 (2 H, s, C*H*_2_), 5.08 (2 H, s, C*H*_2_), 6.69 (2 H, AA′BB′, Ar*H*), 6.77 (1 H, dd, *J*=8.4, 2.3 Hz, Ar*H*), 6.91 (1 H, dd, *J*=8.4, 2.4 Hz, Ar*H*), 6.98 (1 H, s, Ar*H*), 7.19 (1 H, t, *J*=8.6 Hz, Ar*H*), 7.31 (1 H, s, Ar*H*), 7.33–7.45 (5 H, m, Ar*H*), 7.64 (1 H, s, Ar*H*), 7.72 ppm (2 H, AA′BB′, Ar*H*); LRMS (ES+): *m*/*z* (%): 398.9 (100) [*M*+H]^+^; HRMS (ES+): *m*/*z* [*M*+H]^+^ calcd for C_24_H_19_FN_4_O: 399.1616, found: 399.1614.

**4-[(2-Fluoro-4-hydroxybenzyl)(1*H*-imidazol-1-yl)amino]benzonitrile (25 d)**: As general method A using 5 % Pd/C (0.10 g), **25 c** (0.797 g, 2.0 mmol) and THF/MeOH (1:1, 40 mL). The crude product was purified using the CombiFlash *R*_f_ (CH_2_Cl_2_/MeOH) to give the title compound as a white solid (0.50 g, 81 %): mp: 192–194 °C; ^1^H NMR (400 MHz, [D_6_]DMSO): *δ*=4.92 (2 H, s, C*H*_2_), 6.45–6.56 (2 H, m, Ar*H*), 6.69 (2 H, AA′BB′, Ar*H*), 6.97 (1 H, s, Ar*H*), 7.03 (1 H, t, *J*=8.6 Hz, Ar*H*), 7.28 (1 H, s, Ar*H*), 7.60 (1 H, s, Ar*H*), 7.75 (2 H, AA′BB′, Ar*H*), 9.99 ppm (1 H, s, O*H*); ^13^C NMR (100 MHz, [D_6_]DMSO): *δ*=50.4 (CH_2_), 101.9 (C), 102.5 (CH, d), 111.5 (CH), 111.7 (C), 111.8 (C), 113.2 (2×CH), 119.2 (C), 128.0 (CH), 131.7 (CH, d), 133.7 (2×CH), 137.1 (CH), 152.0 (C), 159.1 (C), 161.0 ppm (C, d); ^19^F NMR (376 MHz, [D_6_]DMSO): *δ*=−116.2 ppm (t); LC–MS (ES+): *t*_R_=1.24 min, *m*/*z* (%): 309.0 (100) [*M*+H]^+^.

**4-{[(4-cyanophenyl)(1*H*-imidazol-1-yl)amino]methyl}-3-fluorophenyl sulfamate (25)**: As general method B using ClSO_2_NH_2_ (0.6 m, 5.0 mL), DMA (2 mL) and **25 d** (0.20 g, 0.65 mmol). The crude product was purified using the CombiFlash system (CHCl_3_/acetone) to give the title compound as a white powder (0.21 g, 88 %): ^1^H NMR (400 MHz, [D_6_]DMSO): *δ*=5.08 (2 H, s, C*H*_2_), 6.65 (2 H, AA′BB′, Ar*H*), 6.99 (1 H, s, Ar*H*), 7.02 (1 H, dd, *J*=8.4, 2.0 Hz, Ar*H*), 7.14 (1 H, dd, *J*=10.8, 2.0 Hz, Ar*H*), 7.35 (1 H, s, Ar*H*), 7.42 (1 H, t, *J*=8.4 Hz, Ar*H*), 7.67–7.73 (3 H, m, Ar*H*), 8.10 ppm (2 H, s, 2×C*H*N); ^13^C NMR (100 MHz, [D_6_]DMSO): *δ*=50.7 (CH_2_), 102.2 (C), 109.7 (CH), 113.1 (2×CH), 118.0 (CH), 119.1 (C), 119.2 (CH), 120.6 (C, d), 128.1 (CH), 131.4 (CH), 133.8 (2×CH), 137.2 (CH), 150.6 (C), 151.9 (C), 160.3 ppm (C, d); LC–MS (ES+): *t*_R_=1.10 min, *m*/*z* (%): 388.2 (100) [*M*+H]^+^; HRMS (ES+): *m*/*z* [*M*+H]^+^ calcd for C_17_H_15_FN_5_O_3_S: 388.0874, found: 388.0847.
